# Braiding Braak and Braak: Staging patterns and model selection in network neurodegeneration

**DOI:** 10.1162/netn_a_00208

**Published:** 2021-11-30

**Authors:** Prama Putra, Travis B. Thompson, Pavanjit Chaggar, Alain Goriely

**Affiliations:** Mathematical Institute, University of Oxford, Oxford, United Kingdom; Mathematical Institute, University of Oxford, Oxford, United Kingdom; Mathematical Institute, University of Oxford, Oxford, United Kingdom; Mathematical Institute, University of Oxford, Oxford, United Kingdom

**Keywords:** Braak, Staging, Tau, Network, Neurodegeneration, Diffusion-reaction

## Abstract

A hallmark of Alzheimer’s disease is the aggregation of insoluble amyloid-beta plaques and tau protein neurofibrillary tangles. A key histopathological observation is that tau protein aggregates follow a structured progression pattern through the brain. Mathematical network models of prion-like propagation have the ability to capture such patterns, but a number of factors impact the observed staging result, thus introducing questions regarding model selection. Here, we introduce a novel approach, based on braid diagrams, for studying the structured progression of a marker evolving on a network. We apply this approach to a six-stage ‘Braak pattern’ of tau proteins, in Alzheimer’s disease, motivated by a recent observation that seed-competent tau precedes tau aggregation. We show that the different modeling choices, from the model parameters to the connectome resolution, play a significant role in the landscape of observable staging patterns. Our approach provides a systematic way to approach model selection for network propagation of neurodegenerative diseases that ensures both reproducibility and optimal parameter fitting.

## Author Summary

Network diffusion models of neurodegenerative diseases are a class of dynamical systems that simulate the evolution of toxic proteins on the connectome. These models predict, from an initial seed, a pattern of invasion called staging. The generalized staging problem seeks to systematically study the effect of various model choices on staging. We introduce methods based on braid diagrams to test the possible staging landscape of a model and how it depends on the choice of connectome, as well as the model parameters. Our primary finding is that connectome construction, the choice of the graph Laplacian, and transport models all have an impact on staging that should be taken into account in any study.

## INTRODUCTION

The term ‘neurodegenerative disease’ refers to a family of maladies primarily affecting the brain’s neurons; many neurodegenerative diseases result in cognitive decline and, ultimately, a diagnosis of dementia. A hallmark of such diseases is a large concentration of toxic protein aggregates throughout the brain. These toxic proteins interfere with normal brain functions and are associated with neuronal impairment, neuronal loss, brain atrophy, and overall cognitive decline. Here, we address questions related to model selection for computational studies of Alzheimer’s disease (AD); AD is the most contemporarily prevalent cause of dementia. The practical constraints of clinical AD experiments, most especially in humans, have lead to an interest in the development of mathematical models of pathology evolution, particularly those that can accurately capture large-scale features of AD. Many of these models take advantage of the prion hypothesis which asserts that the progression of proteopathy, in AD, follows from a **prion-like** mechanism.

Prion-like mathematical models can be broadly characterized as either probabilistic (Vogel et al., 2020, 2021) or as continuous (Fornari, Schäfer, Goriely, & Kuhl, 2019a; Raj, Kuceyeski, & Weiner, 2012; Thompson, Chaggar, Kuhl, & Goriely, 2020). Both model types often invoke **nonlinearity** and are typically discretized, and solved, on either a **simplicial mesh** (Weickenmeier, Kuhl, & Goriely, 2018), or on the network defined by the brain’s connectome (Fornari et al., 2019a; Raj et al., 2012; Schäfer, Mormino, & Kuhl, 2020; Thompson et al., 2020). Probabilistic and continuous models have their individual advantages. The former can often reveal unique insights in complex datasets while the mathematical structure of the latter can be rigorously analyzed or, in some simple cases, closed form solutions can be derived. Misfolded protein aggregates, in neurodegenerative diseases, often display structured patterns of progression, and reproducing these staging patterns is a desirable trait for any mathematical model. Here, we will discuss the impact of model selection, for continuous prion-like mathematical models, with respect to an observed structured staging sequence. As a case study, we will consider the network progression of misfolded tau protein (*τ*P) in AD. We compare results to staging patterns motivated by a recent study (DeVos et al., 2018) that uses a six-stage Braak pattern for *τ*P in AD. However, our braid surface approach is generalizable to more complex hierarchical patterns (Cho et al., 2016; Delacourte et al., 1999) and well suited to broader investigations, including applications to the study of staging phenomena in other neurodegenerative diseases.

The primary contribution of this manuscript is a methodical, quantitative study of **model selection** features, as they pertain to structured staging, in the setting of a continuous mathematical model of a prion-like proteopathy processes defined on structural networks. This manuscript introduces two novel investigative tools, braid diagrams and braid surfaces, facilitating this process. Our approach is of practical interest for neurodegenerative diseases for two main reasons. First, there is often a lack of the longitudinal radiotracer data, in which staging would be evident, that would provide for accurate, automatic model selection via, for instance, a **parameter inference** approach. Such studies are important for short-term predictions, but they may make model choices based on a limited number of time points and arrive at a model that does not capture a long-term expected staging progression. Second, we show that there are several other factors of a network neurodegeneration model, beyond mathematical parameters, which are not always considered in the model selection process and can play a significant role in staging. In particular, we show that the choice of **structural connectome** scale, tractography method, thresholding method, **graph Laplacian** weights and, sometimes slight, variation in model parameters can significantly alter the landscape of observed, even accessible, staging patterns.

Our study highlights the nuanced role played by each choice in the network neurodegeneration modeling pipeline, providing important insight, and quantitative tools, that inform model selection. Even though we choose a particular example of an AD staging as a point of study, the methods and observations are general and can be applied to the study of any hierarchical staging process that can appear in the study of neurodegenerative diseases or other dynamical process on networks. The source code, and connectomes, to create the braid diagrams and braid surfaces discussed in the manuscript are freely available online (Putra, Chaggar, Thompson, & Goriely, 2021).

## THEORY AND MODEL

### The Staging Problem

The central problem discussed here is the generalized *staging problem*. We consider a general dynamical system with form

$$\dot{p}\left(t\right)=f\left(t,p;\Theta \right),$$ (1a)

$$p\left(0\right)=p_{0},$$ (1b)

where **p**(*t*) = (*p*_*i*_, …, *p*_*N*_) denotes a normalized and dimensionless quantity, for example, a normalized concentration 0 ≤ *p*_*i*_(*t*) ≤ 1, evolving on a network *G* = (*V*, *E*) with nodes *V* and edge set *E*. The quantity *p*_*i*_(*t*) corresponds to the observed concentration in node *v*_*i*_ and *p*_*i*_(0) is the initial concentration at that node. The quantity **Θ** represents the parameters of the model. In practice, **Θ** represents the parameters of differential Equation 1a in addition to other model selection choices such as the edge weighting scheme, the choice of tractography method, which determines graph connectivity and properties that also influence edge weights, or the edge weight thresholding method used to construct the network *G*. We further assume that the dynamics of the system are such that starting from an initial condition, where all concentrations are taken to vanish except one, the system will evolve asymptotically to a state where all concentrations reach their maximal value. It is the spatiotemporal sequence of invasion that we want to characterize.

Let Ω_*j*_, for *j* = 1, 2, … *J* be a nonoverlapping collection of nodes; that is, Ω_*j*_ and Ω_*k*_ are disjoint subsets of *V* when *j* ≠ *k*. Let *T* ∈ [0, 1] be an arbitrary, but fixed, threshold value. As **p** evolves, according to Equation 1a, the average concentration is computed in each region Ω_*j*_ and the time when Ω_*j*_ first reaches the threshold *T* is recorded. This process produces an ordering of the regions Ω_*j*_ and the ordered sequence Ω_*j*_1__, Ω_*j*_2__, …, Ω_*j*_*J*__ is called an *observed staging pattern*. The *generalized staging problem* is to ascertain the scope of observable staging patterns, subject to variations in **Θ**. The observed staging patterns can then be compared to one or more desirable staging patterns.

### A Continuous Model of τPAD Proteopathy on a Structural Connectome

For our particular application, we model the disease progression on a structural connectome *G* = (*V*, *E*) with node set *V* given by anatomical regions of interest (ROIs) and edge set *E* representing white matter connectivity between these regions. At each node, we define a tau protein concentration, *p*_*i*_, as well as a marker of neurofibirlary tangle (NFT) pathology, *q*_*i*_, and assume the following dynamics:

$$\frac{dp_{i}}{dt}=-\beta \sum_{i=1}^{N}L_{ij}p_{j}+p_{i}\left(1-p_{i}\right),\;i=1,\ldots ,N$$ (2a)

$$\frac{dq_{i}}{dt}=\delta p_{i}\left(1-q_{i}\right),\;i=1,\ldots ,N.$$ (2b)

In Equation 2a the variables *L*_*ij*_ represents the entries of a weighted graph Laplacian **L**, *p*_*i*_ denotes the concentration of seed-competent tau, in node *i*, and *β* a scaled ratio of transmission versus growth (the scaling is chosen so that the coefficient in front of the nonlinear term in Eq. 2a is one). The system Eq. 2a is said to be *growth dominated* when *β* ≪ 1 and *diffusion dominated* when *β* ≫ 1. The seed model Eq. 2a is a network **diffusion-reaction** equation, of **Fisher-Kolmogorov** type, and has been considered in several previous studies (Fornari et al., 2019a; Goriely, Kuhl, & Bick, 2020; Kevrekidis, Thompson, & Goriely, 2020; Schäfer et al., 2020; Weickenmeier, Jucker, Goriely, & Kuhl, 2019; Weickenmeier et al., 2018). In Eq. 2b the variables *q*_*i*_ represent a marker for the local concentration NFT, in an ROI, while *δ* is a coefficient representing an NFT accumulation rate in the presence of *τ*P. This damage model has also been used previously (Goriely et al., 2020; Raj et al., 2012; Raj et al., 2015). Since the available staging patterns, observed in clinical imaging data, do not necessarily have a resolution at the level of a single connectome node, we consider *J* regions Ω_1_, …, Ω_*J*_. In each of these regions, we define

$$P_{j}\left(t\right)=\frac{1}{N}\sum_{i\in V_{j}}p_{i}\left(t\right),\;j=1,\ldots ,J$$ (3)

$$Q_{j}\left(t\right)=\frac{1}{N}\sum_{i\in V_{j}}q_{i}\left(t\right),\;j=1,\ldots ,J,$$ (4)

where *V*_*j*_ is the set of nodes defining region Ω_*j*_ with *N*_*j*_ nodes. In all computations, the first region Ω_1_ is defined as the bilateral entorhinal cortex with *N*_1_ nodes and we choose for initial conditions

$$p_{i}\left(0\right)=\left\{\begin{array}{ll} 0.005/N_{1}, & i\in V_{1}\\ 0, & i\notin V_{1} \end{array}\right.$$ (5)

$$q_{i}\left(0\right)=0,\;i=1,\ldots ,N.$$ (6)

### The Choice of Graph Laplacian

The graph Laplacian used in this work is the *standard graph Laplacian* for an undirected network:

L=D−W. (7)

where **W** is the weighted adjacency matrix associated and **D** is the degree matrix, a diagonal matrix with entries

$$D_{ii}=\sum_{j=1}^{N}W_{ij},\;i=1,\ldots ,N$$

We note that other authors (Abdelnour, Voss, & Raj, 2014; Pandya, Mezias, & Raj, 2017; Pandya et al., 2019; Raj et al., 2012, 2015) have used normalized forms of the graph Laplacian for their models of neurodegenerative disease progression and a natural question for model selection is the choice of a suitable Laplacian for the underlying process.

A parametrized family of graph Laplacians, encompassing both the standard and normalized forms, is given by

$$L_{a,b}=D^{1-a-b}-D^{-a}{WD}^{-b},$$ (8)

where *a*, *b* ∈ [0, 1] and *a* + *b* ≤ 1, the choice *a* = *b* = 0, yielding the standard graph Laplacian and other popular choices include *a* = −1, *b* = 0, *a* = 0, *b* = 1 and the normalized graph Laplacian with *a* = *b* = 1/2. The question is to choose, within this family, a graph Laplacian that respects some desirable properties of the transport it is supposed to model. While it is clear that the dynamics, of tau proteins, do not conserve mass overall, since toxic proteins can be either created through aggregation or removed through clearance, the *transport part* of the model should preserve mass. Otherwise, it would require a model assumption to explain how tau proteins are created or removed simply by being transported from one node to the next and how this creation or removal process depend on the degrees of the two nodes and no other mechanism. Since, there is no known such physical mechanism, we are therefore forced to insist on mass conservation by the diffusion part of the model, whereas growth and clearance should be modeled by other terms in the model as shown in previous studies (Fornari et al., 2019a; Fornari, Schäfer, Goriely, & Kuhl, 2019b; Thompson et al., 2020; Thompson, Meisl, & Goriely, 2021). We show in the Supporting Information S1 that the mass-conservation condition is equivalent to the requirement **1** · **L**_*a*,*b*_ = **0** where **1** = (1, 1, …, 1).

A second condition that we impose on the transport is that transport is driven by difference in concentrations. If the concentration is equal at two nodes, there is no driving force to create an imbalance between these nodes. By analogy with diffusion processes based on Fick’s law, we call this condition the *Fick’s condition*. In terms of the graph Laplacian on an undirected network, it reads simply **L**_*a*,*b*_ · **1** = **0**. We note that there are other possible assumptions when the transport process is viewed as a probabilistic event as shown in Masuda, Porter, and Lambiotte (2017).

We show in Supporting Information S1 that the only possible graph Laplacian, in the class defined by **L**_*a*,*b*_, that satisfies both mass conservation and the Fick’s condition is the standard graph Laplacian **L** = **L**_0,0_. We also show how to generalize this result when regions of different volumes are considered and Fick’s condition suitably generalized.

We want to emphasize that this unavoidable constraint on a physical model of transport does not invalidate previous studies based on the normalized graph Laplacian as these have shown great predictive and explanatory powers when applied to actual patient data. Yet, the choice of using a normalized graph Laplacian is not innocent and should be fully justified as it has some direct implications on the assumptions used in the model, even if these assumptions are not usually given explicitly.

### Graph Laplacian Weightings

We consider networks *G* = (*V*, *E*) based on two families of multiresolution structural connectomes generated from Human Connectome Project (HCP) data (see Methods, Structural connectomes). Each edge, *e*_*ij*_, is associated with two values: the number of fibers *n*_*ij*_ constituting the connection between (anatomical region) nodes *i* and *j*; and the fiber length *ℓ*_*ij*_. We consider the choice of weights as part of the model selection and study three possible weights based on the literature: the length-free weighting (Abdelnour et al., 2014; Raj et al., 2012, 2015), the ballistic weighting (Fornari et al., 2019a, 2019b), and the diffusive weighting (Thompson et al., 2020). The formulas for these three weighting schemes are listed in Table 1.

**Table 1.** Graph Laplacian weightings for model selection

Weighting	Formula for **W**_*ij*_
Length-free weighting (LW)	*n*_*ij*_
Ballistic weighting (BW)	*n*_*ij*_/*ℓ*_*ij*_
Diffusive weighting (DW)	*n*_*ij*_/$\ell_{ij}^{2}$

### Braid Diagrams and Braid Surfaces

A primary contribution of this manuscript is the introduction of braid diagrams and braid surfaces; these powerful tools present a direct and visual assessment of an otherwise complex, nonlinear process evolving in time. We begin by describing a braid diagram. Let *G* = (*V*, *E*) be a fixed network and suppose that Ω_1_, Ω_2_, …, Ω_*J*_ are a fixed set of nonoverlapping node regions. Suppose further that *T*_1_, *T*_2_, …, *T*_*N*_ are (biomarker) threshold values in the unit interval [0, 1]. A braid diagram is a graph whose abscissa is the index of the regions Ω_*j*_ and whose ordinate corresponds to the threshold values *T*_*k*_. As a dynamical system, such as Eq. 2a, evolves on *G*, the time *t*_*j*,*k*_ at which each region, Ω_*j*_, first achieves each threshold, *T*_*k*_, is recorded. If a given threshold is never achieved in a particular region, the recorded time is prescribed as *t*_*j*,*k*_ = ∞. The collection of time values, for a fixed threshold index, establishes an ordering of the regions Ω_*j*_. For each threshold, the ordering can then be visualized as a braid diagram.

**Figure 1.**
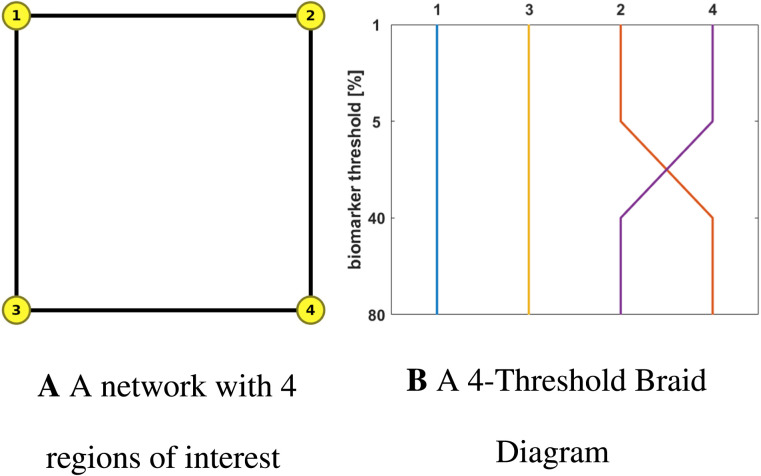
A network with 4 regions of interest (left). A braid diagram (right) generated from an illustrative configuration of fibers (*n*_*ij*_), edge lengths (*ℓ*_*ij*_), DW scheme, and dynamics (*β*).

An illustrative example of a braid diagram is shown in Figure 1. Here, the graph consists of four nodes and the regions are simply Ω_*i*_ = {*i*} for *i* = 1, 2, 3, 4. The threshold values are *T*_1_ = 1%, *T*_2_ = 5%, *T*_3_ = 40%, and *T*_4_ = 80%. Staging was determined from solving Eq. 2a with ln(*β*) = 3.897 and a synthetic weighting matrix:

$$W=\left[\begin{array}{cccc} 0 & 3.125\times 10^{-7} & 5\times 10^{-6} & 0\\ 3.125\times 10^{-7} & 0 & 0 & 1\times 10^{-5}\\ 5\times 10^{-6} & 0 & 0 & 1.5\times 10^{-4}\\ 0 & 1\times 10^{-5} & 1.5\times 10^{-4} & 0 \end{array}\right],$$

which corresponds to the DW scheme (Table 1) for illustrative edge lengths *ℓ*_1,2_ = *ℓ*_2,1_ = 40 and all other edge lengths equal to 20. This simple example shows, for instance, that the regions achieve the threshold *T* = 5% in the order Ω_1_ → Ω_3_ → Ω_2_ → Ω_4_. However, for the threshold *T* = 40%, the observed ordering of regions changes to Ω_1_ → Ω_3_ → Ω_4_ → Ω_2_. These two observed staging patterns can be expressed by the abbreviated notation I → III → II → IV and I → III → IV → II, respectively. A braid diagram is useful for considering observed staging for a fixed set of model parameters, for instance, for a fixed value of *β* in Eq. 2a.

**Figure 2.**
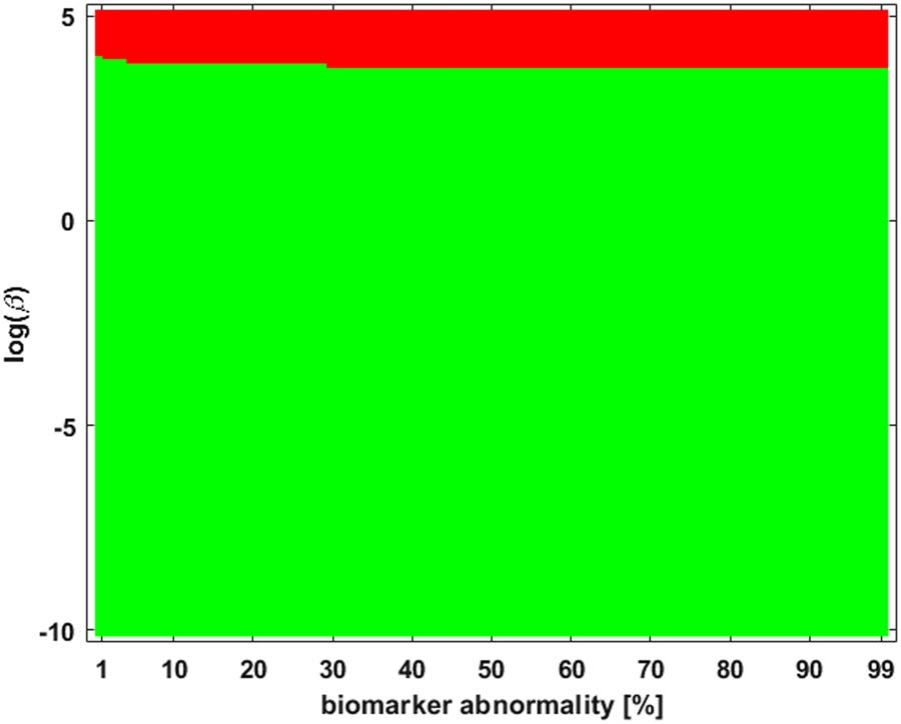
A braid surface showing the *τ*P seed staging dynamics for the simple network of Figure 1 as *β* varies. Two distinct staging patterns are produced for the (diffusive) weights considered.

We are also interested in staging outcomes as the system parameters are varied. This information is contained in a *braid surface* that generalizes a braid diagram. For each possible staging, we assign a color. The braid surface is a two-dimensional plot that assigns for each value of one parameter and one biomarker the corresponding staging color. Computationally, this surface is generated by a simple algorithm. First, one discretizes the continuous values of a parameter, such as *β* in Eq. 2a, of interest. Then, for each discretized parameter, the underlying system (e.g., Eq. 2a) is solved, a braid diagram is constructed and the observed staging pattern is determined for each threshold value; if an observed staging pattern has not yet been encountered, it is added to a list. At the end of the process, every pairing of discrete parameter and threshold has been assigned to an observed staging pattern. The set of observed staging patterns are assigned to colors, and these colors are plotted to visualize the braid surface. The *τ*P seed staging braid surface for the example network, and weighting matrix, of Figure 1 is shown in Figure 2. The green corresponds to the *β*-parameter, and threshold, region where a I → III → II → IV staging pattern is observed while the red corresponds to the parameter region where a I → III → IV → II is produced. The braid surface shows that the staging dynamics, for the four-vertex system with diffusive weights, are simple; we will observe complex staging dynamics on structural connectome graphs of the brain.

### Hierarchical Staging of τP in AD

The problem of the structured staging of *τ*P NFT in AD has been well studied. The most famous study being the seminal work of Braak and Braak (Braak, Alafuzoff, Arzberger, Kretzschmar, & Del Tredici, 2006; Braak & Braak, 1991) that looked at the progression through six regions. The six-region progression view is still in contemporary use (DeVos et al., 2018; Vogel et al., 2020), though some authors have also proposed refinements to ten regions (Delacourte et al., 1999). More recently, the hierarchical progression of flortaucipir tracer has also been studied for progression patterns; authors have studied standardized uptake value ratio (SUVR) progression using a canonical 6-region (Schöll et al., 2016) and an extensive 25-region (Cho et al., 2016) pattern.

Much of the staging literature refers to the evolution of *τ*P NFT or of SUVR quantities, though a recent study (DeVos et al., 2018) has advanced the notion that *τ*P seeds precede NFT pathology in a similar structured manner. The model that we study, that is, Eq. 2a, tracks the progression of both *τ*P seeds (via Eq. 2a) and *τ*P NFT (via Eq. 2b). Therefore, we adopt the staging regions given by DeVos et al. (2018); we will observe how the many, sometimes surprising, aspects of model selection can strongly influence the set of observed regional progressions for a computational model of proteopathic *τ*P staging in AD. The same method can easily be generalized to the study of other staging regions such as those advanced in Cho et al. (2016), Schöll et al. (2016), Vogel et al. (2020), or Delacourte et al. (1999).

## RESULTS

We studied the staging problem for the model (Equation 2a) of *τ*P proteopathy in AD, using the braid surface approach. To study this problem, a five-region adaptation, to match the structural connectome ROI, of the six regions used in DeVos et al. (2018) was adopted and is shown in Table 2.

**Table 2.** Connectome regions used in the staging problem

Stage	Connectome ROI	Stage	Connectome ROI
Region I	entorhinal cortex	Region II	hippocampus
Region III	parahippocampal gyrus	Region IV	rostral anterior cingulate, caudal anterior cingulate
Region V	cuneus, pericalcarine cortex, lateral occipital cortex, lingual gyrus		

We refer to staging progression, determined by a concentration threshold 0% < *T* ≤ 100%, through these five regions with Roman numerals and use the I → II notation. To present the braid surface results, we first enumerate all observed regional staging patterns and assign each one a color as shown in Table 3. From the original point of view (Braak & Braak, 1991) of *τ*P Braak staging, one would expect that the I → II → III → IV → V progression is a likely candidate of interest, at least for NFT. However, based on Alzheimer’s Disease Neuroimaging Initiative (ADNI) data, we also identified three other observed progression sequences that may be of interest for both *τ*P seeds and NFT. The set of identified staging patterns of potential interest are called *Suggested Patterns* and summarized in Table 3 (top).

**Table 3.**
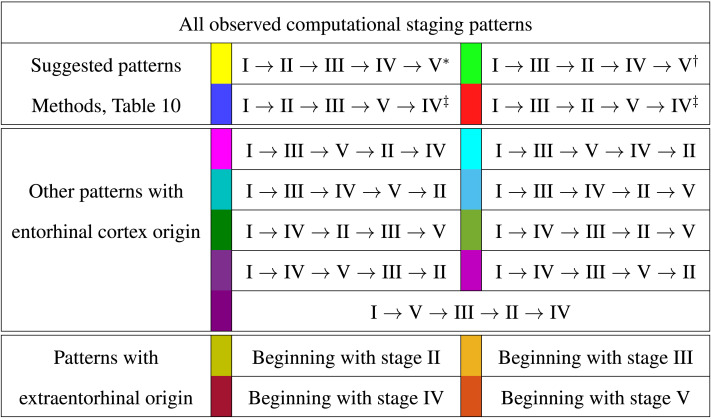
Color coding for braid surfaces

* Progressive computational Braak staging.

† Computational staging suggested by SUVR data.

‡ Additional potential computational staging, *τ*P seeding.

### Connectome Preparation Significantly Alters Observed Staging Patterns

We compared two different types of connectome tractography methods: both *deterministic tractography*, prepared by The PIT Bioinformatics Group (2019) using the MRtrix software, and *probabilistic tractography*, prepared for this study using the FSL software. These generating methods used parcellation ROIs defined by the Lausanne multiresolution atlas and constructed from HCP data. The braid surfaces for the deterministic connectomes and the probabilistic connectomes are, in general, quite different. However, there are some notable similarities. For instance, both connectome types show great sensitivity as the parameter *β* increases to large values. In this diffusion-dominated regime, the observed staging pattern becomes nondistinct with multiple overlap for small changes of parameters. Another common feature is that there are large areas, of *T* and *β* parameter space, where the *τ*P seed staging and *τ*P NFT staging are consistent, meaning that both display the same staging pattern. A final notable similarity is that both connectome types are able to produce the progressive Braak (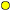) pattern for NFT staging.

**Figure 3.**
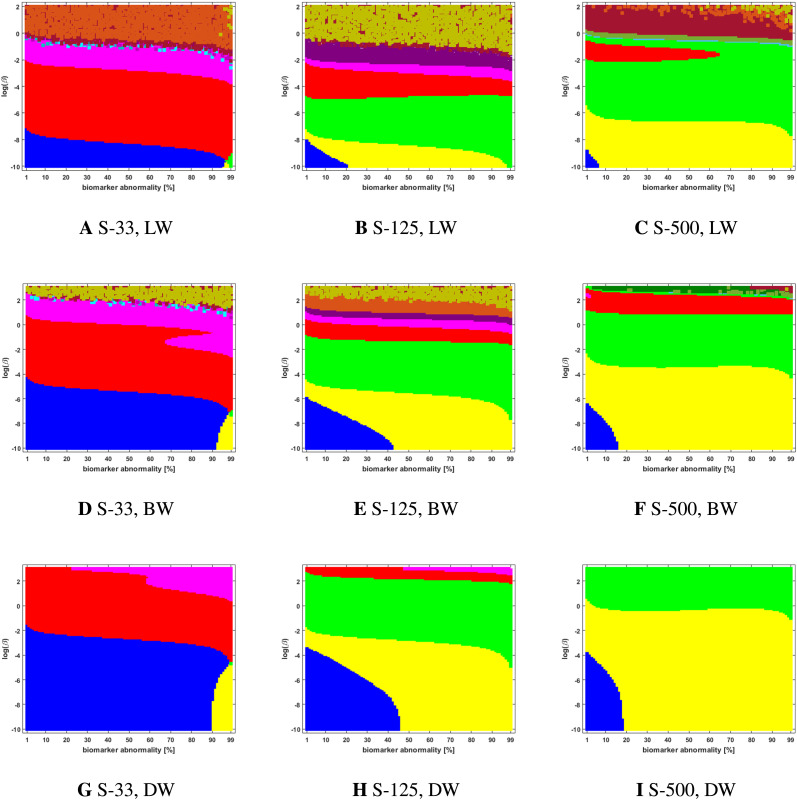
Observed computational (deterministic) connectome *τ*P seed staging. Length-free (top), ballistic (middle), and diffusive (bottom) weighting schemes. The *x*-axis determines the biomarker abnormality threshold 1% < *T* ≤ 100%, and the *y*-axis corresponds to −10 ≤ ln(*β*) ≤ 2 for the parameter *β* in Eq. 2a.

**Figure 4.**
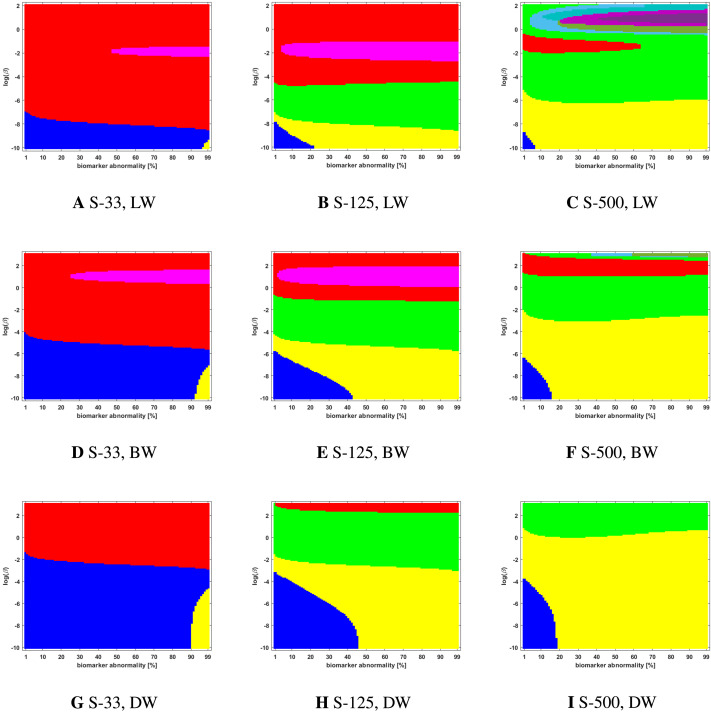
Observed computational (deterministic) connectome *τ*P NFT staging with *δ* = 1 in Eq. 2b. Figure order, axis labels, and axis ranges are identical to those of Figure 3.

**Figure 5.**
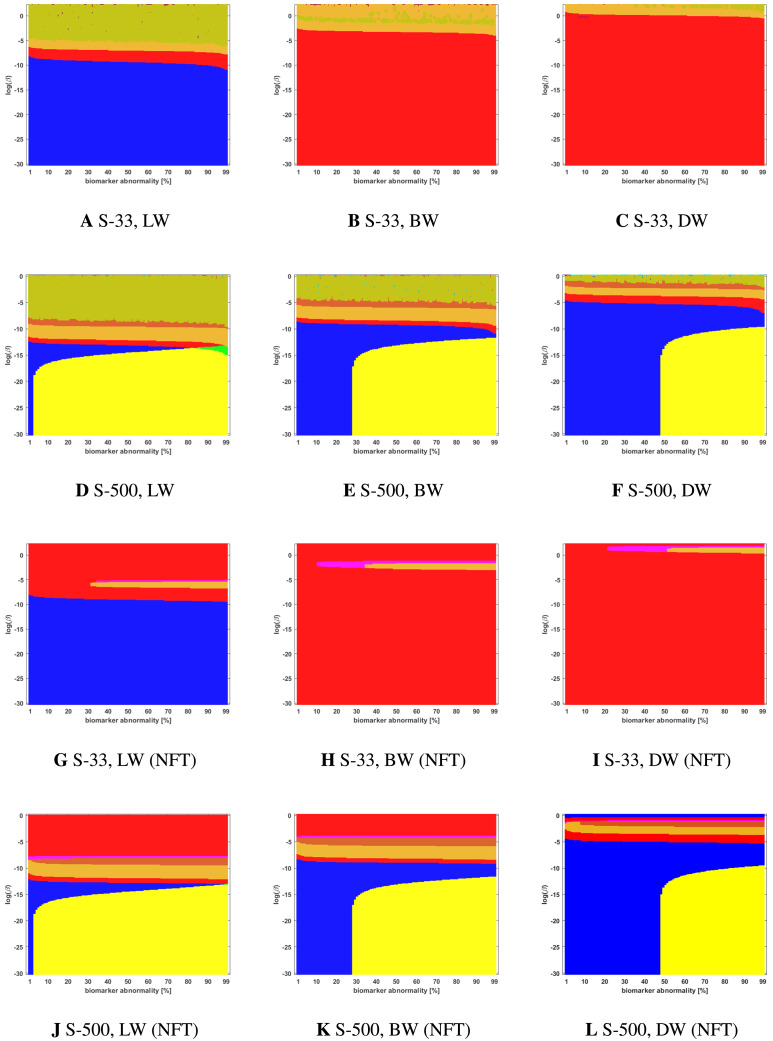
Observed computational (probabilistic) connectome *τ*P seed staging (top two rows) and *τ*P NFT staging (*δ* = 1, bottom two rows). Density filter (DF) thresholding at a threshold of 2 × 10^−1^. The *x*-axis determines the biomarker abnormality threshold 1% ≤ *T* ≤ 100% and the *y*-axis corresponds to −30 ≤ ln(*β*) ≤ 0 for the parameter *β* in Eq. 2a.

**Figure 6.**
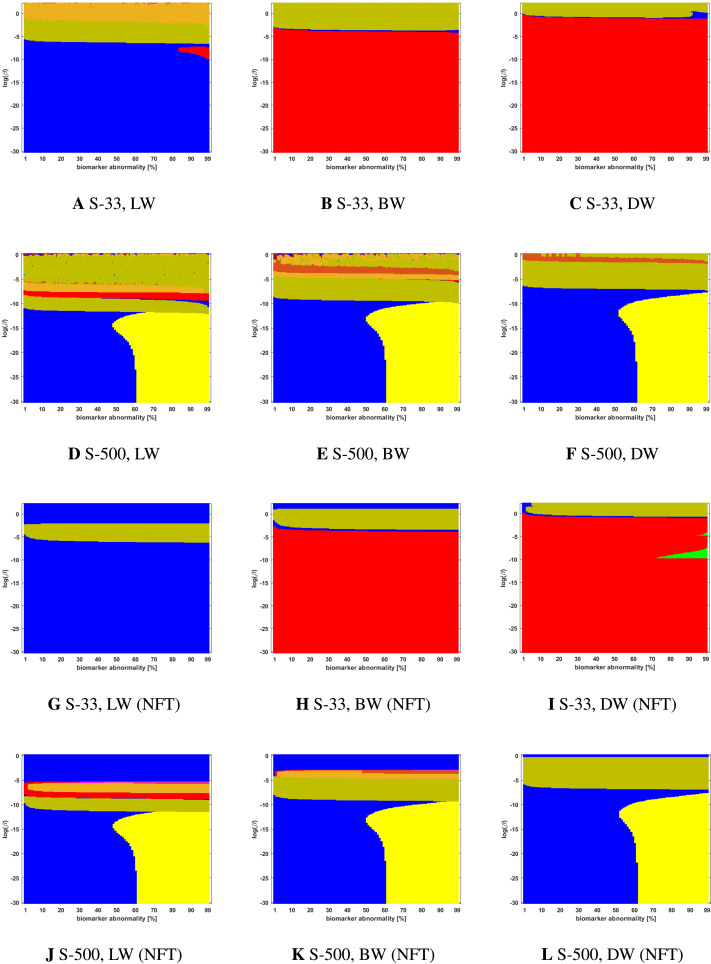
Observed computational (probabilistic) connectome *τ*P seed staging (top two rows) and *τ*P NFT staging (*δ* = 1, bottom two rows). Doubly stochastic thresholding at a threshold of 1 × 10^−2^. Axes coincide with those of Figure 5.

**Figure 7.**
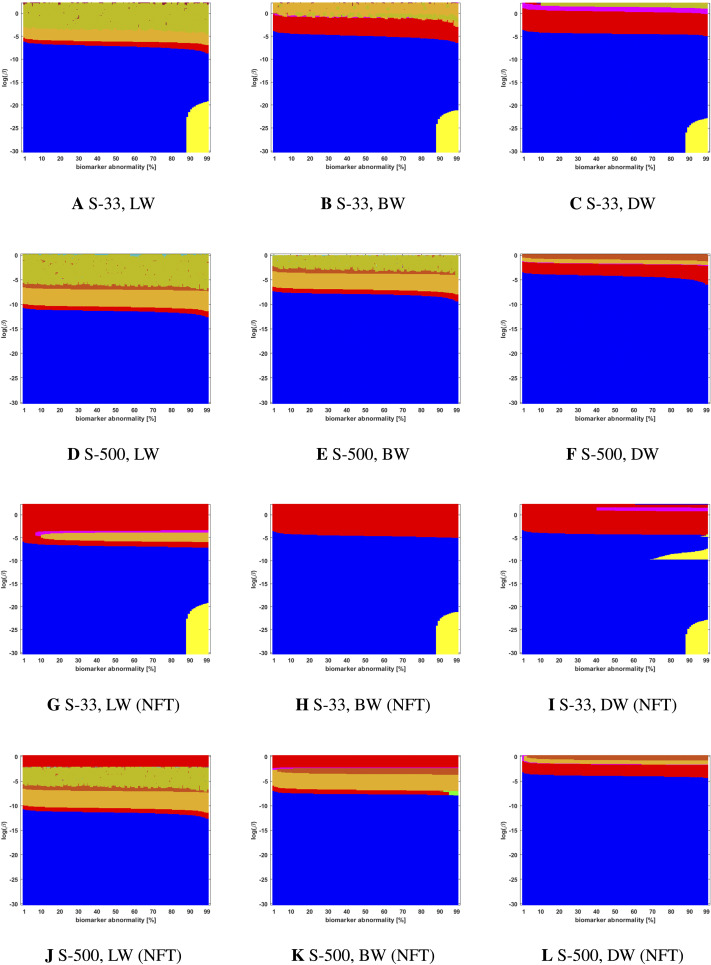
Observed computational (probabilistic) connectome *τ*P seed staging (top two rows) and *τ*P NFT staging (*δ* = 1, bottom two rows). High salience skeleton at a threshold of 1 × 10^−1^. Axes coincide with those of Figure 5.

**Figure 8.**
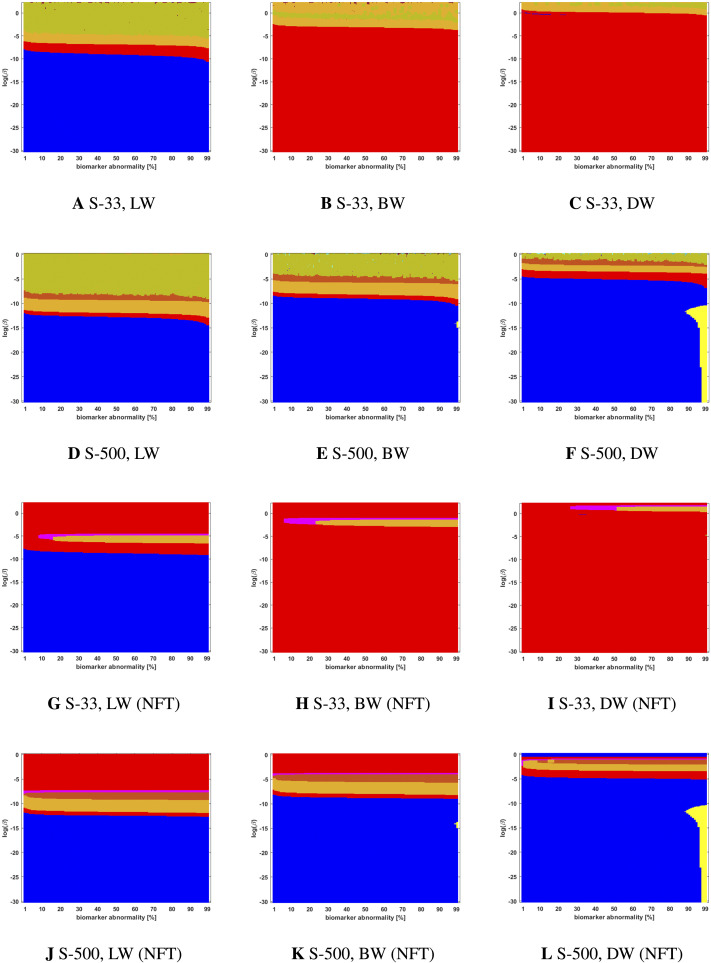
Observed computational (probabilistic) connectome *τ*P seed staging (top two rows) and *τ*P NFT staging (*δ* = 1, bottom two rows). Noise-corrected backbone at a threshold of 2.32. Axes coincide with those of Figure 5.

**Figure 9.**
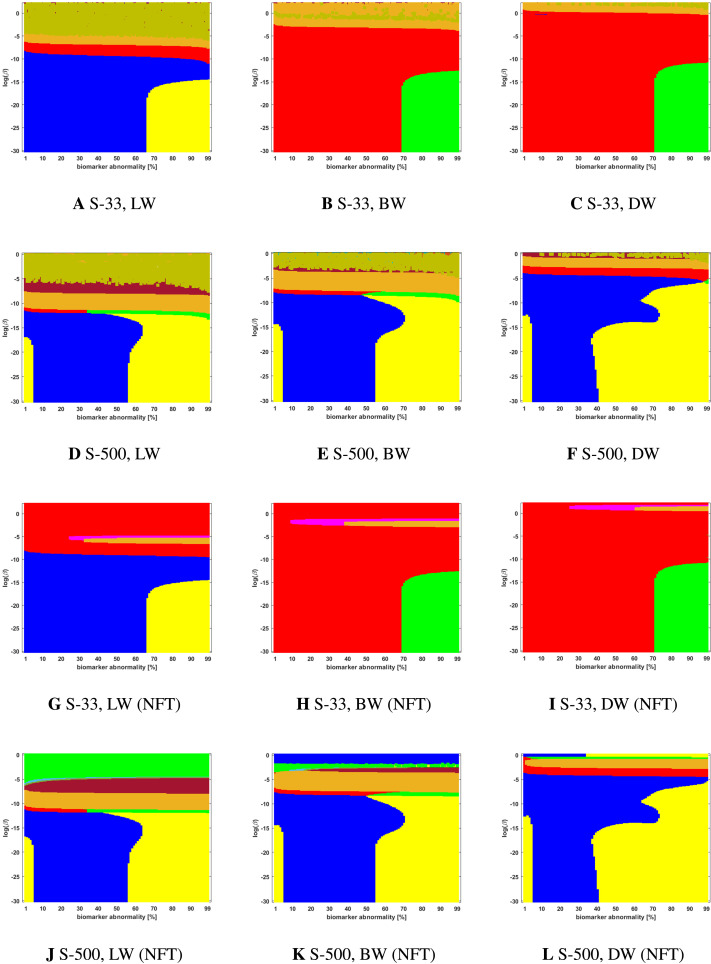
Observed computational (probabilistic) connectome *τ*P seed staging (top two rows) and *τ*P NFT staging (*δ* = 1, bottom two rows). Naive thresholding at a threshold of 1 × 10^−2^. Axes coincide with those of Figure 5.

Despite these similarities, there are also clear differences between the staging behavior observed on the deterministic and probabilistic connectomes. In particular, we examined ADNI SUVR data subject to an ROI selection from a recently published study (DeVos et al., 2018) on *τ*P seeding along the Braak pathway. The SUVR staging pattern suggested (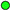) by our analysis appears prominently on the deterministic connectomes (Figure 3 and Figure 4) but is nearly absent on the probabilistic connectome counterparts (Figure 5, Figure 6, Figure 7, Figure 8, and Figure 9). In fact, the deterministic connectomes can express all four suggested staging patterns (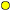, 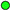, 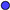, and 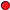) at high resolution for both *τ*P seeds (Figure 3) and NFT (Figure 4) while the probabilistic connectomes almost always express fewer patterns.

We also observed (Figures 5 through 9 and Supporting Information S2) significant qualitative differences in the braid surface landscapes when various thresholding methods were used in preparing the probabilistic connectomes (Table 4). Therefore, we conclude that connectome preparation plays an important role in all downstream results and is an integral part of model selection.

**Table 4.** Thresholding methods for probabilistic connectomes

Connectivity thresholding	Abbreviation	Citation	Results
Disparity filtering	DF	(Serrano, Boguna, & Vespignani, 2009)	Figure 5
Doubly stochastic DF	DS	(Slater, 2009)	Figure 6
High salience skeleton	HSS	(Grady, Thiemann, & Brockmann, 2012)	Figure 7
Noise-corrected backboning	NC	(Coscia & Neffke, 2017)	Figure 8
Naive cutoff	NV	N/A	Figure 9

### The Graph Laplacian Weighting Scheme Modifies Observed Staging Paradigms

Several graph Laplacian weighting schemes have been suggested to model disease dynamics. But, it remains unclear as to which of these weighting choices is most biologically sound. Our results suggest that this choice also affects the observed computational staging patterns in two ways. The first of these concerns *τ*P seed staging within the parameter regime where diffusion takes a more prominent role (i.e., large values of *β*). The move from length-free (LW) to ballistic (BW) weights tends to decrease the surface area where the observed computational staging is sensitive to small changes in the biomarker abnormality threshold; this surface area is again reduced when moving from BW to diffusive (DW) weights. This can be seen in both the deterministic (Figure 3) and probabilistic (Figures 5 through 9, top two rows) braid surface results. In this sense, the length dependence in the weights ‘stabilizes’ the *τ*P seed staging, appearing with an increase in *β*, in Eq. 2a.

The second general observation concerns the prevalence of the progressive Braak (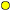) and SUVR-suggested (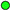) staging patterns. In the former case, the general trend is that the choice of a DW graph Laplacian tends to increase the prevalence of the progressive Braak (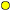) NFT staging (Figure 4 and Figures 6 through 9, bottom two rows). However, we remark that this observation can depend on the choice of thresholding technique as we observed in the case of the density filtered probabilistic connectome where the LW had this effect (Figure 5, bottom two rows and Supporting Information S2 Figure S3, bottom two rows). However, if one were comparing computational results directly to SUVR staging (e.g., Schöll et al., 2016; Vogel et al., 2020), using the ROI of DeVos et al. (2018), the observation is more complex. In the case of deterministic streamlining, the prevalence of the observed SUVR-suggested staging (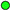) can decrease, within the ranges of *β* we considered, as one moves from LW to BW and again to DW (Figure 4, right-most column). However, we do note that a number of undesirable staging patterns are observed, for LW, when ln(*β*) > 0 on the highest resolution connectome (Figure 4C); thus, extra consideration should be given to the mathematical model parameters in this case. For probabilistic connectomes, the appearance of the SUVR-suggested staging pattern (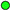) depends more, in general, on thresholding technique and the parcellation resolution; the choice of weights had only a weak impact in this specific regard. Our results suggest that, in the absence of a clear biological impetus for selecting particular graph Laplacian weights, a braid surface analysis can play an important, practical role in selecting amenable weights for a particular study.

### Connectome Resolution Has Variable Effects on Observed Staging

Network neurodegeneration studies have evolved systems, such as Eq. 2a, on both low-resolution (Fornari et al., 2019a; Goriely et al., 2020; Raj et al., 2012, 2015; Schäfer et al., 2020) and high-resolution (Thompson et al., 2020) connectomes. We studied how connectome resolution alters observed computational staging patterns. In the case of connectomes generated with deterministic streamlining, increasing the connectome resolution improved the overall observed staging results. For instance, we see a stabilizing effect on *τ*P seed staging, when ln(*β*) > 0, (columns of Figure 3) with increased connectome resolution. Moreover, the combined area of the progressive and SUVR-suggested staging patterns of interest (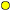 and 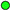, respectively) tend to increase in prominence with increased resolution; conversely, the transposed prefix stagings (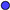 and 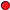) are more pronounced at lower resolutions. This observation also holds for NFT staging (Figure 4).

In the case of probabilistic streamlined connectomes, the thresholding method and the effect of resolution are intertwined (Figures 5 through 9). It is invariably clear that resolution has a pronounced effect on observed staging, but those effects varied depending on the thresholding method. In general, the low-resolution connectomes consisted primarily of a single staging pattern (typically either 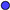 or 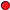) when growth was sufficiently strong (circa ln(*β*) < −5) and increasing the connectome resolution increased heterogeneity in addition to introducing the progressive Braak staging pattern (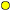).

Together, our results show that connectome resolution plays a deciding role in the landscape of observable staging patterns. In particular, the seemingly ubiquitous practice of the use of low-resolution connectomes may partially explain discrepancies between modeling results and comparisons to imaging data in the latter Braak regions (e.g., IV and V).

### Braid Surfaces for Deterministic Connectomes

Deterministic connectomes were constructed using deterministic streamlining (see Methods). We report the observed staging for both *τ*P seeds (Figure 3) and for NFT (Figure 4). We present these results for the lowest (Scale-33), median (Scale-125), and highest (Scale-500) resolution connectomes in addition to all three graph Laplacian weighting schemes. Both figures summarize results for the model parameter value *β* in the range −10 ≤ ln(*β*) ≤ 2. Further results are reported in the Supporting Information (S2).

### Braid Surfaces for Probabilistic Connectomes

Probabilistic connectomes were constructed from HCP data. We focus on the effects of the parameter *β* and of several connectivity thresholding methods on observed staging results. For brevity, we present only one thresholding level per thresholding method, but other thresholding levels are presented in the Supporting Information (S2).

## DISCUSSION

In the context of studying prion-like spreading of misfolded proteins in AD, different studies have made use of different model choices. For instance, within the class of reaction-diffusion models such as Eq. 2a, several types of graph Laplacian weights have been used; including those that are free of the influence of length (Pandya, Kuceyeski, & Raj, 2017; Raj et al., 2012, 2015), weights that model prion-like transport as a velocity (Fornari et al., 2019a, 2019b), or as diffusive (Thompson et al., 2020) in nature. Various connectome resolutions, for the Lausanne parcellation, have also been used, both low resolution (Fornari et al., 2019a, 2019b; Goriely et al., 2020; Pandya, Kuceyeski, & Raj, 2017; Raj et al., 2012, 2015; Schäfer et al., 2020) and high resolution (Thompson et al., 2020). Several works have compared, or fitted, spreading models to atrophy (Raj et al., 2015) and SUVR data (Pandya, Kuceyeski, & Raj, 2017; Schäfer et al., 2020; Vogel et al., 2020, 2021). However, to our knowledge, a systematic methodical investigation into the general staging problem for AD has not been advanced, even for the simpler class of prion-like progression models such as Eq. 2a.

In this manuscript we have undertaken a methodical investigation of the generalized staging problem for a simple network model of AD; this important problem concerns the ordered progression of a marker of interest propagating over a network. Using an adaptation of a six-stage sequence of ROIs (DeVos et al., 2018) along the Braak pathway, we have systematically investigated how various aspects of model selection, including those mentioned above, may alter the progression of *τ*P seeds and NFT across the connectome. To do so, we have introduced, and applied, the novel tools of braid diagrams and braid surfaces. Though we have focused on a continuous network neurodegeneration model, our method also applies to probabilistic spreading models as well (Iturria-Medina et al., 2014; Vogel et al., 2020).

### Connectome Construction Limits Realizable Staging Patterns

Our findings have several implications for model selection. The most surprising is that the particulars of the connectome itself play an important role in the staging problem. That is, when studying *τ*P progression, the construction of the structural connectome should be considered as an inextricable part of the model. To illustrate this, consider two particular staging patterns: the progressive Braak pathway NFT staging pattern (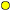) and the staging pattern suggested (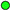) by our ADNI flortaucipir data analysis. It is worth noting that the difference between the progressive Braak (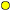) versus the SUVR-suggested (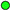) staging is whether a biomarker first appears in the hippocampus (II) or the posterior parahippocampal gyrus (III). In DeVos et al. (2018), the histopathologically identified Braak stage II brains expressed *τ*P seeding in the parahippocampal gyrus (III) at a slightly higher average level than in the hippocampus, potentially explaining the difference between the SUVR-suggested (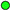) and progressive (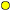) staging patterns. That is, (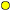) and (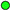) may essentially reflect the same effective staging but from two different points of view. Only the (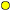) pattern was reliably expressed on the often-used, low-resolution (Scale-33) structural connectome constructed with deterministic streamlining. However, both patterns appeared in the high-resolution (Scale-500) case (Figures 3 and 4, left column vs. right column). Conversely, the high-resolution connectomes generated with probabilistic tractography, and subsequently thresholded for sparsity, almost never produced the (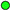) pattern suggested by our ADNI data analysis (Figures 5 through 9, second and fourth rows), though they did produce the progressive staging (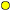). The SUVR pattern (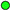) did, however, appear robustly for the Scale-33 connectome when naive thresholding was used (Figure 4). For the Scale-500 naively thresholded connectome, the (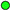) pattern does appear but is only consistent with seed staging in a very small region of parameter and threshold space (see Figure 4, second and last rows). Figure 4 suggests that naive thresholding may be a promising strategy for probabilistic connectomes; however, we found that the (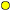) and (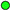) patterns were sensitive to the threshold used (see Supporting Information S2) and were almost completely absent, at both scales, for the nearby thresholds we examined.

The consequences of these observations are nontrivial. For instance, a study comparing the NFT model Eq. 2b to ADNI flortaucipir data on the low-resolution probabilistic tractography connectomes, thresholded with a density filter, would not be capable of achieving the staging pattern (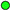) obtained by our SUVR analysis (see Figure 5, third row), invariably leading to a misfit between model and data. Overall, our observations imply that the methods used to construct the structural connectome, for example, the choice of tractography method, parcellation resolution, and any subsequent thresholding, can affect all downstream results and limit the landscape of realizable staging patterns.

### The Graph Laplacian Weight and the Characteristics of Growth versus Diffusion

The graph Laplacian plays a distinctive role in reaction-diffusion models of prion-like propagation such as Eq. 2a; it provides the mechanism for the transport of misfolded proteins over the brain’s structural network. As such, the choice of graph Laplacian weights has varied in the literature; particular weight selections are often motivated by a biological argument or an appeal to data fit. Overall, our results suggest that, while the choice of weights often has a minimal impact on the number of observable staging patterns of interest (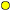, 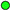, 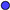, 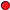), they did affect the prevalence of those patterns, in particular, the diffusive weights (DW) scheme tended to favor the expression of the progressive Braak (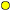) staging pattern over the options of length-free weigt (LW) and ballsitic weights (BW).

**Figure 10.**
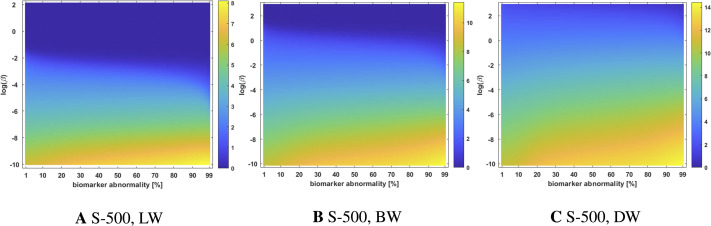
Standard deviation of the staging time series for *τ*P seeds on the high-resolution (S-500) deterministic connectome of Figure 3; all weighting schemes.

In the case of *τ*P seed staging, LW and BW showed significantly more sensitivity, in the observed staging, than the DW scheme as ln(*β*) drew nearer to zero. The staging sensitivity to the weighting scheme can be illustrated by computing the standard deviation of the staging time series. Plots of these standard deviations, for *τ*P seeding on the Scale-500 deterministic connectomes (see Figure 3) are shown in Figure 10. Regions, in Figure 3, where the staging is sensitive to small perturbations in *β* or *T* are precisely those where the standard deviation of the staging time series is low. This implies that the observed sensitivity is a *race condition* where the differences in threshold-attainment time are nearly identical.

As length plays a more dominant role in the weighting scheme, the standard deviation of the staging time series increases, staging patterns are no longer sensitive to small perturbations and *τ*P seed staging becomes pronounced. However, it is not currently known if *τ*P seeds should follow a clear hierarchical progression, though the appearance of *τ*P seeds have been shown to proceed NFT pathology (DeVos et al., 2018). In the case of NFT staging, the DW scheme generally promoted the progressive Braak pathway NFT staging pattern (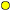) across all connectomes. This pattern, however, was not the SUVR-suggested (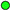) pattern according to our analysis. It is worth noting that the patient data necessary to achieve more statistically significant results, for latter stages, was not present (see Methods, Data preparation and Methods, Identifying additional computational staging patterns of interest). Moreover, as we mentioned previously, the II → III versus III → II paradigm, differentiating the progressive staging (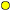) from the SUVR-suggested staging (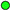), may be related to the prevalence of seeds (DeVos et al., 2018) in the posterior parahippocampus at histopathological Braak classification stage II. Nevertheless, our results suggest that direct comparisons to flortaucipir data, at least for the ROI we considered, may be best conducted (see Figures 3F and 4F) with the BW scheme and on high-resolution connectomes created with deterministic streamlining and with a choice of mathematical parameters given by −2 ≲ ln(*β*) ≲ 1.

Conversely, if a progressive Braak pattern (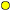) is desirable, this can most reliably be achieved, in general, on high-resolution connectomes with the DW scheme, sufficiently dominant growth (i.e., *β* sufficiently small) and a typical biomarker threshold of at least 50%. As a point of practice, this should be checked with a braid surface analysis, as we did observe that thresholding methods can perturb this trend on the probabilistic connectomes that we considered. For example, Figure 5 shows that the density filter threshold favors the LW and BW schemes over the DW scheme for the progressive staging (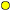); likewise, Figure 7 shows that the high-salience scheme more readily expresses the progressive staging (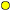) on lower resolution connectomes.

### Conclusion

Investigations of *τ*P staging have been carried out histopathologically (Braak et al., 2006; Braak & Braak, 1991; Delacourte et al., 1999), using SUVR (Cho et al., 2016; Schöll et al., 2016), and in fitting models of spreading to SUVR data (Schäfer et al., 2020; Vogel et al., 2020, 2021). As computational power is readily available, the prion-like hypothesis has enabled a new generation of mathematical models facilitating the latter types of studies. However, it is currently not known how various model selection choices may promote, or limit, the expression of particular staging patterns. We have found that every aspect of model selection can alter the overall progression of *τ*P staging. This includes parcellation resolution, tractography method, choice of thresholding method, choice of threshold, graph Laplacian weights, and the mathematical model parameters. We have shown that the the generalized staging problem couples the global topology of the structural connectome to the mathematical model governing the local dynamics of a biomarker evolving on that network and that comprehensive model selection cannot, in general, be ensured from a decoupled perspective. We have introduced braid diagrams and braid surfaces as a means to investigate the complex staging landscape, and changes thereto, for this coupled problem and to observe the implications of model selection choices.

The generalized staging problem in AD reveals the need for more experimental data and poses new theoretical questions. We propose that further study into the progression patterns of *τ*P seed staging, extending the results of DeVos et al. (2018), could promote a more reliable selection of parameter ranges for the balance of growth and diffusion (*β*) for *τ*P seeds and, due to the consistency we observed, for *τ*P NFT. Moreover, an increased number of SUVR data for patients in the latter Braak stages would help to belay uncertainty in determining the progression patterns suggested by data studies. Mathematically, the study of the coupling between the connectome topology, the weighted distances between ROIs, and the model parameters is a promising candidate for theoretical research.

## METHODS

### Structural Connectomes

**Figure 11.**
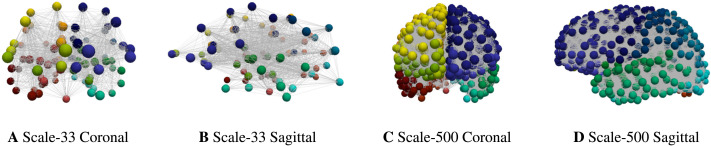
Lowest (left) and highest (right) connectome resolutions. Node colors signify the 83 disjoint anatomical parcellation regions.

Braid surfaces have been introduced to facilitate model selection, in the context of staging patterns developing on two sets of undirected, multiresolution structural connectomes; both sets of connectomes were originally constructed from the data of participants in the HCP. All of the structural connectomes considered in this manuscript were constructed using the Lausanne multiresolution atlas parcellation (Daducci et al., 2012) with five levels of potential resolution: the coarsest scale (Scale-33), three intermediate scales (Scale-60, Scale-125, Scale-250), and a fine scale (Scale-500). The edges, at all scales, include information regarding the number of fibers (*n*_*ij*_) and fiber length (*ℓ*_*ij*_) associated to each edge (*e*_*ij*_) connecting region *i* to region *j*. The first set of connectomes (see Figure 11) were constructed using MRtrix (Kerepesi, Szalkai, Varga, & Grolmusz, 2017; Tournier, Calamante, & Connelly, 2012) and a deterministic streamlining with 20,000 streamlines and randomized seeding. These connectomes span five scales; the lowest and highest scales are shown in Figure 11. These connectomes are publicly available, as the dataset named ‘Full set, 426 brains, 20,000 streamlines’, from The PIT Bioinformatics Group (2019).

The second set of connectomes were constructed for this study in order to assess degrees of certitude regarding observations for the deterministic connectomes mentioned above. The Connectome Mapping Toolkit (Daducci et al., 2012) was used to parcellate a high-resolution MNI reference template; the FSL (Jenkinson, Beckmann, Behrens, Woolrich, & Smith, 2012; Smith et al., 2004; Woolrich et al., 2009) PROBTRACKX algorithm was employed, for the probablistic tractography, with 10,000 streamlines per voxel. The sparsity of the resulting connectivity matrices was low (approximately 7–12%); these matrices are commonly thresholded before use in computational models. To study the effect of thresholding, we considered five different thresholding techniques in our comparative analysis. The thresholding techniques used are summarized in Table 4, and a description of the method can be found in the corresponding citation. The naive thresholding method removes edge *e*_*ij*_ if the corresponding connectivity coefficient (*n*_*ij*_) is below a prescribed threshold value.

For comparison, we have constructed connectomes for the lowest (Scale-33, 50 patients) and highest scale (Scale-500, 25 patients) resolutions of the Lausanne multiresolution atlas. The braid surface source code, and the full set of thresholded connectomes, used in this study is available online (Putra et al., 2021).

### A Staging for *τ*P Seeds on Structural Connectomes

We have discussed several hierarchical staging patterns for *τ*P progression in AD. As our mathematical model accounts for both *τ*P seeds and *τ*P NFT, we selected a six-stage model that has been related to both quantities (DeVos et al., 2018). These six regions, along the Braak pathway, are enumerated in Table 5.

**Table 5.** Anatomical staging regions for *τ*P seeds as reported in DeVos et al. (2018)

Region I	entorhinal cortex	Region II	hippocampus
Region III	posterior parahippocampal gyrus	Region IV	anterior cingulate
Region V	visual association cortex	Region VI	primary visual cortex

The regions in Table 5 can be mapped to anatomical ROI of the Lausanne atlas used in the construction of the structural connectomes for this study. We therefore adapt the stages of Table 5 to the Lausanne atlas by considering the staging process of Table 2. The primary difference is that stages V and VI, of Table 5, are combined into a single terminal stage and approximated by the presence of deposition in the cuneus, pericalcarine cortex, lateral occipital cortex, and lingual gyrus.

### Staging Sequences for *τ*P Pathology on Computational Connectomes

**Figure 12.**
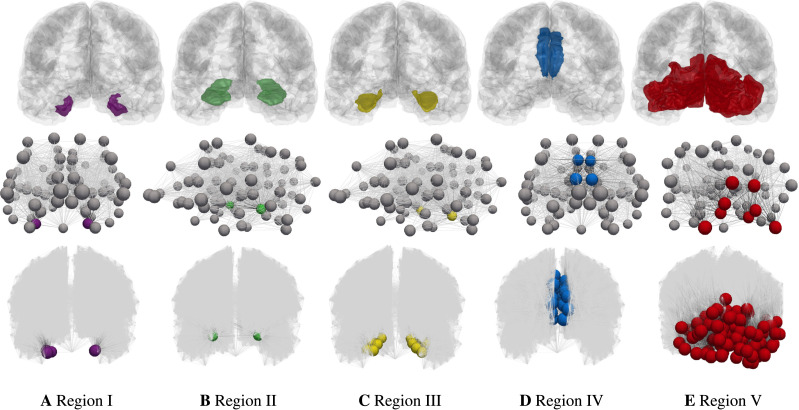
Connectome Braak staging regions, Ω_*i*_. Anatomical regions in a glass brain (top). Coarsest connectome resolution (Scale-33 parcellation, middle) and finest resolution (Scale-500 parcellation, bottom, additional nodes suppressed) connectomes.

In order to assess the implications of various model parameters on observed computational staging patterns, thus facilitating model selection, a choice of preferable staging patterns must be identified. We select five collections of nodes, Ω_*i*_ for *i* = 1, 2, …, 5, such that Ω_*k*_ is the set of nodes of the computational connectomes whose anatomical ROI labels are given by the *k*^th^ stage of Table 2; thus Ω_1_ contains all nodes labeled as belonging to the left and right entorhinal cortices, etc. The staging regions, Ω_*i*_, for the coarsest (top row) and finest (bottom row) connectome resolutions are shown in Figure 12.

The work of DeVos et al. (2018) assessed *τ*P seed staging along the Braak tau pathway and found that tau seeding precedes the presence of NFT pathology. A clear candidate for both *τ*P seed and NFT staging is therefore the progressive regional staging I → II → III → IV → V. To assess other potential staging sequences we used flortaucipir (AV-1451) data from the ADNI; the data was fully preprocessed at the Helen Wills Neuroscience Institute at the University of California, Berkeley, and the compiled results are freely available through the ADNI website. The preprocessed dataset contains 1,184 records; each record corresponds to a subject structural MRI scan and tau PET scan, and the preprocessing steps are explained in the companion document available through the ADNI website.

#### Data preparation

The Berkeley preprocessing pipeline is as follows: each patient T1 MRI was segmented using FreeSufer 7.1.1; each subject’s normalized-intensity flortaucipir scan was coregistered to their bias-corrected T1 image; partial volume effects were corrected for using the geometric transfer matrix approach (Baker, Maass, & Jagust, 2017; Baker et al., 2017); and mean flortaucipir uptake is reported, in the dataset, for each region of the FreeSurfer segmentation. We further normalized each subject’s regional SUVR score using the inferior cerebellar gray matter as the reference region, as suggested in the dataset documentation.

We proceeded to create two datasets using this preprocessed dataset. For the first set, we computed the volume-weighted SUVR average for each patient record over the whole brain to compute a global score (GS) and for each region of Table 2. Thus, each ADNI patient visit record was assigned a global volume-averaged SUVR score along with five volume-averaged regional scores corresponding to the regions of Table 2. We refer to this dataset as the ‘base dataset’.

We then created a second dataset we refer to as the ‘partitioned dataset’ by following the methodology of Schöll et al. (2016). An overview of the creation process follows: First, the GS was used as a decision variable, in a conditional inference tree (CIT) algorithm. Second, the CIT algorithm was used to compare the GS to the BraakV regional volume-weighted SUVR, producing a GS threshold. This GS threshold was used to separate patient records into two groups who differed significantly based on their BraakV score. The BraakV patient records were then moved, out of the full dataset, into a new data file. This process was then repeated, once again applying the CIT algorithm and using the GS as a decision variable, but now comparing to the BraakIV regional volume-weight SUVR score, thus creating a BraakIV group of patient records, and so on. This process resulted in five separate files, that is, the partitioned dataset, corresponding to a patient record classification according to the regions of Table 2. The thresholds produced by the CIT algorithm can be seen in Table 6. Our application of CITs follows the identical approach of previous authors (Schöll et al., 2016), in a similar SUVR study, to categorize each patient record into the discrete groups corresponding to a predefined set of regional stages.

**Table 6.** Conditional inference-based partitioning of ADNI flortaucipir data into the stages of Table 2

Stage group classification	Volume-averaged global SUVR (GS)	Subject records in group
I	1.266 ≤ GS	36
II	1.266 < GS ≤ 1.392	248
III	1.392 < GS ≤ 1.52	443
IV	1.52 < GS ≤ 2.365	416
V	GS > 2.365	41

#### Identifying additional computational staging patterns of interest

Our next goal was to see if ADNI flortaucipir data may suggest alternatives to the progressive I → II → III → IV → V (computational) Braak-like sequence, in particular for NFT due to the binding of flortaucipir to paired helical filaments. Our objective here is not to rigorously study hierarchical SUVR behavior as in Cho et al. (2016). Rather, our goal is to conduct a simple inquiry as to whether the ADNI data may suggest alternative staging patterns of potential interest for the computational staging problem and model selection, especially as it pertains to observed NFT staging.

Our first investigation used the partitioned dataset. First, we checked that the CIT algorithm separated the data in such a way that all of regional mean SUVR values, across all possible group-region pairings, were significantly (*p* < 0.05) different, using a Welch’s *t* test; this was the case, as expected by an application of CIT-based separation. Next, we computed the Pearson correlation between each stage group’s primary stage and all other stages within that group; the results are reported in Table 7.

**Table 7.** Pearson correlation between a stage group’s primary region and all other regions within the group

Patient group and region	Connectome Braak regional SUVR correlation				
	Region I	Region II	Region III	Region IV	Region V
Group I, Region I	1.0*	0.26	0.53*	0.14	0.04
Group II, Region II	0.34*	1.0*	0.31*	0.14*	−0.29*
Group III, Region III	0.69*	0.32*	1.0*	0.06	−0.09
Group IV, Region IV	0.11*	0.28*	0.32*	1.0*	−0.13*
Group V, Region V	−0.13	−0.03	0.16	−0.08	1.0*

*Note*. A * denotes a statistically significant correlation (*p* < 0.05) between regions.

Assuming that overt NFT pathology originates in region I, Table 7 suggests that staging sequences with I → III may also be of interest for NFT. Assuming that III proceeds I, the next suggested progression would be III → II. This observation suggests that sequences starting with the prefix I → III → II could be of interest alongside the more progressive stage prefix I → II → III. Finally, likely due to the sparsity of patient records assigned to Group V, none of the correlations of Group V were significant.

Our next investigation uses the base dataset. The primary motivation was to ensure that patient gender and age were not confounding our partitioning-based strategy. Starting with the base dataset, we cross-referenced ADNI patient data and augmented the base set with the age, at the time of scan, in addition to the patient’s gender. We then used regression models covarying for age and gender. We compared each regional volume-averaged SUVR score to that of the other regions; the results are shown in Table 8. Evidence for influence was evaluated using standardized factor scores when the influence was significant. Region III was most affected by Region I (*R*^2^ = 0.775, *B* = 0.613, *p* < 0.001), further supporting interest in the I → III staging prefix. Region II was also most influenced by Region I (*R*^2^ = 0.486, *B* = 0.486, *p* < 0.001) but at a lower value of both *B* and *R*^2^. Removing Region I from the model decreased the variational fidelity (*R*^2^) of the Region III model by 24.8% and the Region II model fidelity by 13.6%; in the latter case, the Region II score was most affected by the Region III score (*R*^2^ = 0.42, *B* = 0.5). Supposing that pathology begins in the entorhinal cortex (Region I), these observations suggest, again, that a I → III → II prefix is also of interest for evaluating computational staging.

**Table 8.** Regression models, covarying for age and gender, of regional influence

Regression model	Influential model region(s)	Model *R*^2^	*B*	*p*-value
Region II	Region I	0.486	0.49	*p* < 0.001
	Region III	0.42	0.5	*p* < 0.001
Region III	Region I	0.78	0.61	*p* < 0.001
	Region IV	0.58	0.39	*p* < 0.001
	Region II		0.36	*p* < 0.001
Region IV	Region III	0.43	0.75	*p* < 0.001
Region V	Region III	0.22	0.38	*p* < 0.001

Next, we take a second look at the staging suffix options IV → V versus V → IV. To do so, we regressed the Region IV and Region V scores in terms of the first three regional scores, once more covarying for age and gender. The Region IV model identified Region III as the most influential (*R*^2^ = 0.43, *B* = 0.75, *p* < 0.001). The Region V model was only marginally explained by the data (*R*^2^ = 0.22), but the most influential factor was, again, the Region III score (*B* = 0.38, *p* < 0.001), but with a substantially lower influence than in the Region IV model. These latter observations suggest that a IV → V may be more desirable than a V → IV suffix. We have therefore corroborated, and extended, the conclusions suggested by the partitioning strategy (Table 7).

Next, we corroborate our previous SUVR findings by following a similar approach to Cho et al. (2016) and considering z-scores, computed from the base dataset, for the regions defined in Table 2. First, all of the regional mean SUVR scores were individually standardized. Similar to Cho et al. (2016), we considered a particular region to be ‘involved’ in a fixed patient record if that region’s Z score satisfied *Z* ≥ 2. We then selected all patient records with an involved Region I (entorhinal cortex). Continuing within this subset of data, we counted the frequency of all other involved regions and computed their mean z score. The results are reported in Table 9. Once more, we see that progression sequences starting with I → III → II may also be of computational interest and, further, that the staging suffix IV → V appears more preferable, for SUVR, than the V → IV alternative.

**Table 9.** Spreading order of *τ*P (SUVR) for Region I involved patient records

Region	% Involved	Z
Region I	100.0	3.24
Region III	55.1	2.55
Region II	46.94	1.81
Region IV	22.45	1.43
Region V	22.45	1.17

Finally, we compared the ADNI data findings, above, to the results for *τ*P seeds reported in DeVos et al. (2018) where Braak II brains (classified postmortem) also showed a slight average increase of *τ*P seeds in physiological region III (i.e., region III, Table 5) over that of physiological region II; further suggesting that a computational I → III → II pattern may be of interest for *τ*P seeds. If one considers post-mortem Braak classification as a pseudo-time then, from DeVos et al. (2018, Fig. 3), it is tempting to suggest a computational I → III → II → IV → V pattern of interest for *τ*P seeds. However, the differential between seeding levels in the latter stages is slight. In summary, ADNI SUVR data, and results from DeVos et al. (2018), suggest a broader set, beyond the choice of a strictly ascending progression, of potentially interesting computational staging patterns for the choice of regions in Table 2. These staging patterns are reported in Table 10. We note that either more late stage data (i.e., Group V, Table 6), a finer SUVR staging sequence, such as that of Cho et al. (2016), or both is needed to ascertain a higher degree of certainty in the latter *τ*P computational staging patterns for AD.

**Table 10.** Computational staging patterns of potential interest for *τ*P seeds and NFT

Computational staging patterns (see Table 2)		
Canonical late staging	I → II → III → IV → V*	I → III → II → IV → V^†^
Uncertain late staging	I → II → III → V → IV^‡^	I → III → II → V → IV^‡^

*Note*. *Progressive Braak staging, ^†^Suggested by SUVR data, ^‡^Additional connectome computational stagings of potential interest pertaining to uncertain *τ*P seed staging prefixes.

## ACKNOWLEDGMENTS

Data used in the preparation of this article were obtained from the Alzheimer’s Disease Neuroimaging Initiative (ADNI) database (adni.loni.usc.edu). The ADNI was launched in 2003 as a public-private partnership, led by Principal Investigator Michael W. Weiner, MD. The primary goal of ADNI has been to test whether serial magnetic resonance imaging (MRI), positron emission tomography (PET), other biological markers, and clinical and neuropsychological assessment can be combined to measure the progression of mild cognitive impairment (MCI) and early Alzheimer’s disease. For up-to-date information, see www.adni-info.org. A complete listing of ADNI investigators can be found at: https://adni.loni.usc.edu/wp-content/uploads/how_to_apply/ADNI_Acknowledgement_List.pdf

## SUPPORTING INFORMATION

Supporting information for this article is available at https://doi.org/10.1162/netn_a_00208.

## AUTHOR CONTRIBUTIONS

Prama Putra: Conceptualization; Formal analysis; Investigation; Software; Visualization; Writing – review & editing. Travis B. Thompson: Conceptualization; Data curation; Formal analysis; Investigation; Methodology; Project administration; Software; Supervision; Visualization; Writing – original draft; Writing – review & editing. Alain Goriely: Conceptualization; Formal analysis; Funding acquisition; Methodology; Project administration; Resources; Software; Supervision; Writing – original draft; Writing – review & editing. Pavanjit Chaggar: Data curation; Formal analysis; Software; Writing – review & editing.

## FUNDING INFORMATION

Alain Goriely, Engineering and Physical Sciences Research Council (https://dx.doi.org/10.13039/501100000266), Award ID: EP/R020205/1. Travis B. Thompson, John Fell Fund, University of Oxford (https://dx.doi.org/10.13039/501100004789), Award ID: 000872. Pavanjit Chaggar, Engineering and Physical Sciences Research Council (https://dx.doi.org/10.13039/501100000266), Award ID: EP/L016044/1.

## TECHNICAL TERMS

Prion-like: A prion-like biological agent is one that replicates an undesirable state through an autocatalytic process.
Nonlinearity: A nonlinearity is any mathematical expression that does not satisfy *f* (*a***x** + **y**) = *af* (**x**) + *f* (**y**) where *a* is a real number and **x** and **y** are input vectors.
Simplicial mesh: A, often complex, mesh whose elements are triangles (2D) or tetrahedra (3D); widely used to solve partial differential equations on complex domains.
Model selection: The process of arriving at the set of features that determine the model used to study a process of interest.
Parameter inference: Typically refers to mathematical approaches to deduce distributions for the parameters of a model, from observed data, using statistical paradigms such as Bayesian inference.
Structural connectome: A structural connectome is a graph representation *G* = (*V*, *E*) of the brain where the vertices *V* are anatomical regions of interest and the edges represent connections between regions of interest via axonal bundles.
Graph Laplacian: The graph Laplacian is a matrix representation of a transport process on a, often weighted and undirected, network graph.
Diffusion-reaction: A partial differential equation describing the simultaneous diffusion and reactive production of an agent through a medium.
Fisher-Kolmogorov: A class of diffusion-reaction equations, that admit traveling wave solutions, where the reaction term satisfies a set of conditions. This class includes Fisher’s equation as a special case.

## References

[bib1] Abdelnour, F., Voss, H., & Raj, A. (2014). Network diffusion accurately models the relationship between structural and functional brain connectivity networks. Neuroimage, 90, 335–347. https://doi.org/10.1016/j.neuroimage.2013.12.039, PubMed: 24384152 24384152 10.1016/j.neuroimage.2013.12.039 PMC3951650

[bib2] Baker, S., Lockhart, S., Price, J., He, M., Huesman, R., Schonhaut, D., … Jagust, W. (2017). Reference tissue-based kinetic evaluation of 18F-AV1451 for tau imaging. Journal of Nuclear Medicine, 58(2), 332–338. https://doi.org/10.2967/jnumed.116.175273, PubMed: 27587706 27587706 10.2967/jnumed.116.175273 PMC5288744

[bib3] Baker, S., Maass, A., & Jagust, W. (2017). Considerations and code for partial volume correcting [(18F)]-AV-1451 tau PET data. Data Brief, 15, 648–657. https://doi.org/10.1016/j.dib.2017.10.024, PubMed: 29124088 29124088 10.1016/j.dib.2017.10.024 PMC5671473

[bib4] Braak, H., Alafuzoff, I., Arzberger, T., Kretzschmar, H., & Del Tredici, K. (2006). Staging of Alzhimer disease-associated neurofibrillary pathology using paraffin sections and immunocytochemistry. Acta Neuropathologica, 112(4), 389–404. https://doi.org/10.1007/s00401-006-0127-z, PubMed: 16906426 16906426 10.1007/s00401-006-0127-z PMC3906709

[bib5] Braak, H., & Braak, E. (1991). Neuropathological stageing of alzheimer-related changes. Acta Neuropathologica, 82(4), 239–259. https://doi.org/10.1007/BF00308809, PubMed: 1759558 1759558 10.1007/BF00308809

[bib6] Cho, H., Choi, J., Hwang, M., Kim, Y., Lee, H.-M., Lee, H.-S., … Lyoo, C. (2016). In vivo cortical spreading pattern of tau and amyloid in the Alzheimer disease spectrum. Annals of Neurology, 80(2), 247–258. https://doi.org/10.1002/ana.24711, PubMed: 27323247 27323247 10.1002/ana.24711

[bib7] Coscia, M., & Neffke, F. (2017). Network backboning with noisy data. In 2017 IEEE 33rd International Conference on Data Engineering (ICDE) (pp. 425–436). 10.1109/ICDE.2017.100

[bib8] Daducci, A., Gerhard, S., Griffa, A., Lemkaddem, A., Cammoun, L., Gigandet, X., … Thiran, J.-P. (2012). The Connectome Mapper: An open-source processing pipeline to map connectomes with MRI. PLoS One, 7(12), e48121. https://doi.org/10.1371/journal.pone.0048121, PubMed: 23272041 23272041 10.1371/journal.pone.0048121 PMC3525592

[bib9] Delacourte, A., David, J.-P., Sergeant, N., Buee, L., Wattez, A., Vermersch, P., … Di Menza, C. (1999). The biochemical pathway of neurofibrillary degeneration in aging and Alzheimer’s disease. Neurology, 52(6), 1158–1158. https://doi.org/10.1212/wnl.52.6.1158, PubMed: 10214737 10214737 10.1212/wnl.52.6.1158

[bib10] DeVos, S.-L., Corjuc, B.-T., Oakley, D.-H., Nobuhara, C.-K., Bannon, R., Chase, A., … Hyman, B. (2018). Synaptic tau seeding precedes tau pathology in human Alzheimer’s disease brain. Frontiers in Neuroscience, 12, 267. https://doi.org/10.3389/fnins.2018.00267, PubMed: 29740275 29740275 10.3389/fnins.2018.00267 PMC5928393

[bib11] Fornari, S., Schäfer, A., Goriely, A., & Kuhl, E. (2019a). Prion-like spreading of Alzheimer’s disease within the brain’s connectome. Journal of the Royal Society Interface. https://doi.org/10.1098/rsif.2019.0356, PubMed: 31615329 10.1098/rsif.2019.0356 PMC6833337 31615329

[bib12] Fornari, S., Schäfer, A., Goriely, A., & Kuhl, E. (2019b). Spatially-extended nucleation-aggregation-fragmentation models for the dynamics of prion-like neurodegenerative protein-spreading in the brain and its connectome. Journal of Theoretical Biology. https://doi.org/10.1016/j.jtbi.2019.110102, PubMed: 31809717 10.1016/j.jtbi.2019.110102 31809717

[bib13] Goriely, A., Kuhl, E., & Bick, C. (2020). Neuronal oscillations on evolving networks: Dynamics, damage, degradation, decline, dementia, and death. Physical Review Letters, 125(12), 128102. https://doi.org/10.1103/PhysRevLett.125.128102, PubMed: 33016724 33016724 10.1103/PhysRevLett.125.128102

[bib14] Grady, D., Thiemann, C., & Brockmann, D. (2012). Robust classification of salient links in complex networks. Nature Communications, 3(864). https://doi.org/10.1038/ncomms1847, PubMed: 22643891 10.1038/ncomms1847 22643891

[bib15] Iturria-Medina, Y., Sotero, R., Toussant, P., & Alan, C. (2014). Epidemic spreading model to characterize misfolded proteins propagation in aging and associated neurodegenerative disorders. PLoS Computational Biology, 10(11), e1003956. https://doi.org/10.1371/journal.pcbi.1003956, PubMed: 25412207 25412207 10.1371/journal.pcbi.1003956 PMC4238950

[bib16] Jenkinson, M., Beckmann, C., Behrens, T., Woolrich, M., & Smith, S. (2012). FSL. NeuroImage, 62, 782–790. https://doi.org/10.1016/j.neuroimage.2011.09.015, PubMed: 21979382 21979382 10.1016/j.neuroimage.2011.09.015

[bib17] Kerepesi, C., Szalkai, B., Varga, B., & Grolmusz, V. (2017). The braingraph.org database of high resolution structural connectomes and the brain graph tools. Cognitive Neurodynamics, 11, 483–486. https://doi.org/10.1007/s11571-017-9445-1, PubMed: 29067135 29067135 10.1007/s11571-017-9445-1 PMC5637719

[bib18] Kevrekidis, P., Thompson, T., & Goriely, A. (2020). Anisotropic diffusion and traveling waves of toxic proteins in neurodegenerative diseases. arXiv:2007.02421.

[bib19] Masuda, N., Porter, M. A., & Lambiotte, R. (2017). Random walks and diffusion on networks. Physics Reports, 716, 1–58. 10.1016/j.physrep.2017.07.007

[bib20] Pandya, S., Kuceyeski, A., & Raj, A. (2017). The brain’s structural connectome mediates the relationship between regional neuroimaging biomarkers in Alzheimer’s disease. Journal of Alzheimer’s Disease, 55(4), 1639–1657. https://doi.org/10.3233/JAD-160090, PubMed: 27911289 10.3233/JAD-160090 27911289

[bib21] Pandya, S., Mezias, C., & Raj, A. (2017). Predictive model of spread of progressive supranuclear palsy using directional network diffusion. Frontiers in Neurology, 8, 692. https://doi.org/10.3389/fneur.2017.00692, PubMed: 29312121 29312121 10.3389/fneur.2017.00692 PMC5742613

[bib22] Pandya, S., Zeighami, Y., Freeze, B., Dadar, M., Collins, D., & Raj, A. (2019). Predictive model of spread of Parkinson’s pathology using network diffusion. NeuroImage, 192, 178–194. https://doi.org/10.1016/j.neuroimage.2019.03.001, PubMed: 30851444 30851444 10.1016/j.neuroimage.2019.03.001 PMC7180066

[bib23] Putra, P., Chaggar, P., Thompson, T., & Goriely, A. (2021). Oxford Mathematical Brain Modelling group: Braid surface Matlab source code and master connectome graphs, Github, https://github.com/OxMBM/Connectome-Staging

[bib24] Raj, A., Kuceyeski, A., & Weiner, M. (2012). A network diffusion model of disease progression in dementia. Neuron, 73(6), 1204–1215. https://doi.org/10.1016/j.neuron.2011.12.040, PubMed: 22445347 22445347 10.1016/j.neuron.2011.12.040 PMC3623298

[bib25] Raj, A., LoCastro, E., Kuceyeski, A., Tosun, D., Relkin, N., & Weiner, M. (2015). Network diffusion model of progression predicts longitudinal patterns of atrophy and metabolism in Alzheimer’s disease. Cell Reports, 10(3), 359–369. https://doi.org/10.1016/j.celrep.2014.12.034, PubMed: 25600871 25600871 10.1016/j.celrep.2014.12.034 PMC5747552

[bib26] Schäfer, A., Mormino, E., & Kuhl, E. (2020). Network diffusion modeling explains longitudinal tau PET data. Frontiers in Neuroscience, 14, 1370. https://doi.org/10.3389/fnins.2020.566876, PubMed: 33424532 10.3389/fnins.2020.566876 PMC7785976 33424532

[bib27] Schöll, M., Lockhart, S., Schonhaut, D., O’Neil, J., Janabi, M., Ossenkoppele, R., … Jagust, W. (2016). PET imaging of tau deposition in the aging human brain. Neuron, 89(5), 971–982. https://doi.org/10.1016/j.neuron.2016.01.028, PubMed: 26938442 26938442 10.1016/j.neuron.2016.01.028 PMC4779187

[bib28] Serrano, M., Boguna, M., & Vespignani, A. (2009). Extracting the multiscale backbone of complex weighted networks. PNAS, 106(16), 6483–6488. https://doi.org/10.1073/pnas.0808904106, PubMed: 19357301 19357301 10.1073/pnas.0808904106 PMC2672499

[bib29] Slater, P. (2009). A two-stage algorithm for extracting the multiscale backbone of complex weighted networks. PNAS, 106(26), E66. https://doi.org/10.1073/pnas.0904725106, PubMed: 19549841 19549841 10.1073/pnas.0904725106 PMC2705523

[bib30] Smith, S., Jenkinson, M., Woolrich, M., Beckmann, C., Behrens, T., Johansen-Berg, H., … Matthews, P. (2004). Advances in functional and structural MR image analysis and implementation as FSL. NeuroImage, 23(S1), 208–219. https://doi.org/10.1016/j.neuroimage.2004.07.051, PubMed: 15501092 10.1016/j.neuroimage.2004.07.051 15501092

[bib31] The PIT Bioinformatics Group. (2019). Connectomes: The Braingraph.org public website. Braingraph.org. https://braingraph.org/cms/download-pit-group-connectomes/

[bib32] Thompson, T., Chaggar, P., Kuhl, E., & Goriely, A. (2020). Protein-protein interactions in neurodegenerative diseases: A conspiracy theory. PLoS Computational Biology, 16(10), e1008267. https://doi.org/10.1371/journal.pcbi.1008267, PubMed: 33048932 33048932 10.1371/journal.pcbi.1008267 PMC7584458

[bib33] Thompson, T., Meisl, G., & Goriely, A. (2021). The role of clearance mechanisms in the kinetics of toxic protein aggregates involved in neurodegenerative diseases. Journal of Chemical Physics, 154, 125101. https://doi.org/10.1063/5.0031650, PubMed: 33810689 10.1063/5.0031650 33810689

[bib34] Tournier, J.-D., Calamante, F., & Connelly, A. (2012). MRtrix: Diffusion tractography in crossing fiber regions. International Journal of Imaging Systems and Technology, 22(1), 53–66. 10.1002/ima.22005

[bib35] Vogel, J., Itturia-Medina, Y., Strandberg, O., Smith, R., Levitis, E., Evans, A., & Hansson, O. (2020). Spread of pathological tau proteins through communicating neurons in human Alzheimer’s disease. Nature Medicine, 11(2612). https://doi.org/10.1038/s41467-020-15701-2, PubMed: 32457389 10.1038/s41467-020-15701-2 PMC7251068 32457389

[bib36] Vogel, J., Young, A., Oxtoby, N., Smith, R., Ossenkoppele, R., Strandberg, O., … Hansson, O. (2021). Four distinct trajectories of tau deposition identified in Alzheimer’s disease. Nature Communications, 27, 871–881. https://doi.org/10.1038/s41591-021-01309-6, PubMed: 33927414 10.1038/s41591-021-01309-6 PMC8686688 33927414

[bib37] Weickenmeier, J., Jucker, M., Goriely, A., & Kuhl, E. (2019). A physics-based model explains the prion-like features of neurodegeneration in Alzheimer’s disease, Parkinson’s disease, and amyotrophic lateral sclerosis. Journal of the Mechanics and Physics of Solids, 124, 264–281. 10.1016/j.jmps.2018.10.013

[bib38] Weickenmeier, J., Kuhl, E., & Goriely, A. (2018). Multiphysics of prionlike diseases: Progression and atrophy. Physical Review Letters, 121(15), 158101. https://doi.org/10.1103/PhysRevLett.121.158101, PubMed: 30362787 30362787 10.1103/PhysRevLett.121.158101

[bib39] Woolrich, M., Jbabdi, S., Patenaude, B., Chappell, M., Makni, S., Behrens, T., … Smith, S. (2009). Bayesian analysis of neuroimaging data in FSL. NeuroImage, 45, S173–S186. https://doi.org/10.1016/j.neuroimage.2008.10.055, PubMed: 19059349 19059349 10.1016/j.neuroimage.2008.10.055

