# Supplementary material for: Braiding Braak and Braak: Staging patterns and model selection in network neurodegeneration
Source: Netw Neurosci. 2021 Nov 30;5(4):929–56. doi: 10.1162/netn_a_00208 (PMC8746141; doi:10.1162/netn_a_00208)
Supplement: Supplementary file 1 [file netn-05-929-s001.pdf]

# Supplementary Information S1
# Diffusion reaction systems and the conservative graph Laplacian

## A continuous diffusion-reaction model

Our present focus is on non-linear diffusion-reaction models. The typical scalar is form for such models is

$$\frac{\partial p}{\partial t} = \rho \nabla \cdot (\mathbf{K} \nabla p) + R(p), \tag{1}$$

where $p$ is the concentration of misfolded $\tau$P, $R(p)$ represents the non-linear reaction term for the model, $\mathbf{K}$ is a (symmetric) matrix diffusion tensor and $\rho$ is an effective diffusion constant; when the diffusion is isotropic, $\mathbf{K}$ is a multiple of the identity matrix and $\nabla \cdot (\mathbf{K} \nabla p) = \rho \Delta p$ is the usual continuous Laplacian operator. In the brain, axonal white-matter bundles heavily bias the diffusion of extracellular fluid; leading to a transversely anisotropic diffusion tensor [1] with general form

$$\mathbf{K} = d_{\perp} \mathbf{I} + \left(d_{||} - d_{\perp}\right) \gamma \otimes \gamma, \tag{2}$$

where $\mathbf{I}$ is the identity matrix, $d_{\perp} \ll d_{||}$ and $\gamma$ is a unit vector oriented along the fiber bundle. We now note two properties of (1). First, the form of the diffusive term, $\nabla \cdot (\mathbf{K} \nabla p)$, in (1) is a direct result of Fick's law of diffusion. Second, in the absence of any explicit mass exchange terms, such as production or clearance, or specific reaction terms, the model (1) conserves mass; a feature that should be consistent in a discretized version of the problem.

In the main manuscript, we study the generalized staging problem, on networks, of the simplest, single-species model for the prion-like propagation of $\tau$P proteopathy which promotes both prion-like propagation and growth; this Fisher-Kolmogorov-Petrovsky-Piskunov (Fisher-KPP) model has been used in several previous investigations [1, 2, 3, 4, 5]. This model allows for a direct study of staging in the presence of prion-like growth (due to the reaction terms) and diffusive transport. The Fisher-KPP model is defined by setting $R(p) = \alpha p(1-p)$ to obtain

$$\frac{\partial p}{\partial t} = \rho \nabla \cdot (\mathbf{K} \nabla p) + \alpha p(1-p), \tag{3a}$$

$$p(x, 0) = p_0(x), \tag{3b}$$

$$K \nabla p \cdot n = 0 \quad \text{on } \partial\Omega, \tag{3c}$$

where $0 \leq \alpha$ determines the local growth rate, potentially varying smoothly in space, and $p_0(x)$ is the initial seeding of $\tau$P. We will be particularly interested in the observed staging behavior, along the Braak pathway, of network discretizations of (3) with respect to the relative size of the parameters $\rho$ and $\alpha$; the Fisher-KPP model has two asymptotic regimes:

| | |
|---|---|
| Diffusion-dominated | $\max_{\Omega} \alpha \ll \rho$, |
| Growth-dominated | $\rho \ll \min_{\Omega} \alpha$. |

## Connectome discretizations of diffusion-reaction models

The problem now is to find a suitable discretizations of the spreading process by using the network structure of the brain. While some mouse models are sufficiently precise to establish directionality and hence build a directed connectome [6, 7], parallel evidence for directed flow within the human brain is lacking. Moreover, there is no available directional human structural connectome and it has been shown, theoretically, that directionality has very little appreciable impact on the overall spreading dynamics [5]. We therefore consider an undirected connected network $G = (V, E)$ with $|V| = N \geq 3$ nodes and $|E| = M \geq 2$ edges. We further assume that the network is weighted with positive weights $\mathbf{W}_{ij} = \mathbf{W}_{ji}$ at an edge joining node $i$ to node $j$. Weights are assumed to be zero if two nodes are not connected, strictly positive if two nodes are connected and the graph is assumed to be free of self-loops (i.e. $\mathbf{W}_{ii} = 0$) at each vertex. The weighting matrix $\mathbf{W}$ therefore defines an $N \times N$ symmetric *weighted adjacency matrix* on the undirected graph $G$. For the remainder of the discussion, we presume that the graph $G$ represents a structural connectome of the human brain. That is, the vertices of $G$ represent regions of interest (ROI), determined by a choice of parcellation, and the edges of $G$ represent the white matter fiber bundle connectivity between the ROIs.

Within the brain, the prion-like propagation of misfolded proteins is dominated by its transport along the axonal fiber bundles. It is therefore natural to consider propagation along edges of the structural connectome graph, $G$, discussed above. To do so, we replace scalar fields, such as $p$, $\partial_t p$, $\alpha(x)$ etc, with regional (ROI) averages, denoted by $p_i$, $\dot{p}_i$, or $\alpha_i$ etc, and replace the continuous diffusion operator $\nabla \cdot (\mathbf{K} \nabla p)$ with a graph Laplacian. This type of discretization strategy can be considered as motivated by, for instance, finite volume methods [8, 9] applied, in space, to (1) with a simple quadrature, such as the midpoint rule, for estimating the (spatial) integral of the non-linear term, $R(p)$, on a mesh whose cell faces are constructed orthogonal to the fiber directions. A natural discretization of (1) is therefore

$$\frac{\mathrm{d}p_i}{\mathrm{d}t} = -\sum_{j=1}^{N} \mathcal{L}_{ij} p_j + R(p_i), \tag{4}$$

where $p_i$ is the concentration at each ROI $i$ and $\mathcal{L}$ is the $N \times N$ graph Laplacian. The standard graph Laplacian has the form

$$\mathcal{L} = \mathbf{D} - \mathbf{W} \tag{5}$$

where $\mathbf{W}$ is a weighted adjacency matrix that codifies the connectivity between vertices of the graph $G$ and $\mathbf{D}$ is the diagonal degree matrix defined by

$$\mathbf{D}_{ii} = \sum_{j=1}^{N} W_{ij}$$

The graph Laplacian matrix can also be normalized, which we will discuss in the following section. Using the graph Laplacian, the full connectome graph discretization of (3) is

$$\frac{\mathrm{d}p_i}{\mathrm{d}t} = -\rho \sum_{j=1}^{N} L_{ij} p_j + \alpha p_i (1 - p_i), \tag{6a}$$

$$p_i(0) = p_{i,0}, \quad i = 1, 2, \ldots, N, \tag{6b}$$

The continuous Neumann boundary condition (3c) is enforced, at the network level, by ensuring that the graph Laplacian robustly conserves mass; mass conserving forms of the graph Laplacians are the topic of the next section. We close by mentioning that (6) can be rescaled in time to produce the model considered in the primary manuscript; namely,

$$\frac{\mathrm{d}p_i}{\mathrm{d}t} = -\beta \sum_{j=1}^{N} L_{ij} p_j + p_i(1 - p_i), \tag{7a}$$

$$p_i(0) = p_{i,0}, \quad i = 1, 2, \ldots, N, \tag{7b}$$

where $\beta = \rho/\alpha$.

## The many graph Laplacians

In this section we consider the properties of mass conservation and Fick's law with respect to the form of the graph Laplacian matrix $\mathcal{L}$. To motivate this investigation, we consider (7a) without the additional reaction term; that is,

$$\frac{\mathrm{d}\mathbf{p}}{\mathrm{d}t} = -\mathcal{L} \cdot \mathbf{p}, \tag{8}$$

where $\mathbf{p} = (p_1, \ldots, p_n)$ is the (column) vector of concentrations. First, assume that each ROI has the same volume; we will consider the case of varying volumes momentarily. Mass conservation, which we state as condition (C1) below, requires that

$$\sum_{i=1}^{n} \frac{\mathrm{d}p_i}{\mathrm{d}t} \equiv \mathbf{1} \cdot \frac{\mathrm{d}\mathbf{p}}{\mathrm{d}t} = 0, \tag{9}$$

where $\mathbf{1} = (1, \ldots, 1)$ is the one vector. Using (8), this condition implies

$$\mathbf{1} \cdot \mathcal{L} \cdot \mathbf{p} = 0, \tag{10}$$

which must be true for all $\mathbf{p}$. Hence we have the condition

$$\text{(C1):} \quad \mathbf{1} \cdot \mathcal{L} = \mathbf{0}, \tag{11}$$

where $\mathbf{0} = (0, \ldots, 0)$ is the null vector. Fick's condition, which we state as (C2) below, states that in the absence of a concentration gradient, there is no transport; this statement is equivalent to the condition that

$$\text{(C2):} \quad \mathcal{L} \cdot \mathbf{1} = \mathbf{0}. \tag{12}$$

When the graph Laplacian is symmetric, conditions (C1) and (C2) are mathematically equivalent. In addition to (C1) and (C2) we also consider a *robustness condition*, stated as

$$\text{(C3): conditions (C1) and (C2) hold for all weighted adjacency matrices } \mathbf{W} \tag{13}$$

Condition (C3) states, in particular, that variations in the weighted adjacency matrix should not incur a loss of the fundamental physical principles reflected by conditions (C1) and (C2). This is an important practical requirement, as graph Laplacian weightings for structural connectomes are often derived from tractography algorithms, determining the number of fibers and their lengths, which may report different values when different software packages are used.

**A family of Laplacians** The standard graph Laplacian (5) has been used in several modeling studies [3, 10, 11] of network neurodegeneration. Normalized forms of the graph Laplacian have also been used to study network spreading of proteopathy [12], the relationship between structural and functional connectivity [13], atrophy in Alzheimer's disease [14], and pathology in both supranuclear palsy [15], and Parkinson's disease [16]. The *normalized graph Laplacian* used in these works is

$$\mathbf{L}_{1/2,1/2} = \mathbf{I} - \mathbf{D}^{-1/2}\mathbf{W}\mathbf{D}^{-1/2}. \tag{14}$$

Other possible Laplacian matrices can be defined and have been used in various studies such as spectral clustering, including

$$\mathbf{L}_{1,0} = \mathbf{I} - \mathbf{D}^{-1}\mathbf{W} = \mathbf{D}^{-1}\mathbf{L}, \tag{15}$$

$$\mathbf{L}_{0,1} = \mathbf{I} - \mathbf{W}\mathbf{D}^{-1} = \mathbf{L}\mathbf{D}^{-1}, \tag{16}$$

which motivates the introduction of the family

$$\mathbf{L}_{a,b} = \mathbf{D}^{1-a-b} - \mathbf{D}^{-a}\mathbf{W}\mathbf{D}^{-b} = \mathbf{D}^{-a}\mathbf{L}\mathbf{D}^{-b}, \tag{17}$$

with $a, b \in [0, 1]$ and $a + b \leq 1$. The standard graph Laplacian corresponds to the choice $a = b = 0$. That is, $\mathbf{L} = \mathbf{L}_{0,0}$ and we note that since the network is connected, the degree of each ROI (node) is strictly positive and the inverse of $\mathbf{D}$ is well defined. We can now state the main result of this section

**Proposition 1.** *Suppose $G = (V, E)$ is a connected undirected network with weighted adjacency matrix $\mathbf{W}$. Then, the standard graph Laplacian $\mathbf{L}_{0,0} = \mathbf{L}$ is the only member of the family* (17) *that simultaneously satisfies conditions (C1), (C2) and (C3).*

*Proof.* From the identities

$$\mathbf{D} \cdot \mathbf{1} = \mathbf{1} \cdot \mathbf{D} = \mathbf{W} \cdot \mathbf{1} = \mathbf{1} \cdot \mathbf{W} = \mathbf{d}, \tag{18}$$

It follows that

$$\mathbf{L} \cdot \mathbf{1} = \mathbf{1} \cdot \mathbf{L} = \mathbf{0}. \tag{19}$$

Hence the standard Laplacian $\mathbf{L}$ satisfies conditions (C1) and (C2). Condition (C3) is satisfied by the fact that these relationships do not depend on the form of $\mathbf{W}$. Hence, changes in the weight will not affect either condition.

Next, we show that $\mathbf{L}_{0,0}$ is the only member of the family (17) satisfying all of (C1), (C2) and (C3). Let $a, b \in [0, 1]$, with $a + b \leq 1$, be arbitrary but fixed with at least one of $a > 0$ or $b > 0$. First, consider conditions (C2) and (C3). From (17), and (C2) we have

$$\mathbf{L}_{a,b} \cdot \mathbf{1} = \mathbf{D}^{1-a-b} \cdot \mathbf{1} - \mathbf{D}^{-a}\mathbf{W}\mathbf{D}^{-b} \cdot \mathbf{1} = \mathbf{0}.$$

The $i^{\text{th}}$ component of the above equation is

$$d_i^{1-a-b} - \sum_{j=1}^{N} \frac{W_{ij}}{d_i^a d_j^b} = \frac{d_i}{d_i^a d_i^b} - \sum_{j=1}^{N} \frac{W_{ij}}{d_i^a d_j^b} = 0.$$

Multiplying through by $d_i^a$ and using the definition of $\mathbf{D}$ in terms of $\mathbf{W}$ yield

$$\sum_{j=1}^{N}\left(\frac{W_{ij}}{d_i^b}-\frac{W_{ij}}{d_j^b}\right)=\sum_{j=1}^{N}\frac{W_{ij}(d_j^b-d_i^b)}{d_j^b d_i^b}=0. \tag{20}$$

Since this identity must be respected for all undirected connected graphs (condition (C3)), it must be independent of $\mathbf{W}$, hence we must have

$$d_j^b-d_i^b=0, \tag{21}$$

for all pairs $(i,j)$. This condition is satisfied by either $b=0$ or $d_i=d_j$. If $d_i=d_j$ for all ROIs (nodes) and since there are at least 3 ROIs (nodes) in the network, one can change the weight of a single edge connected to one of the ROI, say $i$, but not the other by adding an arbitrarily small amount $0<\epsilon\ll 1$ to that weight, with the effect of changing $d_i$ but not $d_j$. Hence, the equality $d_i=d_j$ cannot hold under the robustness assumption and we conclude that $b=0$ is the only condition for which (C2) and (C3) hold simultaneously.

Next we consider the conditions (C1) and (C3). From (17), and (C1) we have

$$\mathbf{1}\cdot\mathbf{L}_{a,b}=\mathbf{1}\cdot\mathbf{D}^{1-a-b}-\mathbf{1}\cdot\left(\mathbf{D}^{-a}\mathbf{W}\mathbf{D}^{-b}\right)=\mathbf{0}.$$

The $j^{\text{th}}$ component of the column vector corresponding to the above equation states that

$$d_j^{1-a-b}-\sum_{i=1}^{N}\frac{w_{ij}}{d_j^a d_i^b}=\frac{d_j}{d_j^a d_j^b}-\sum_{i=1}^{N}\frac{w_{ij}}{d_i^a d_j^b}=0,$$

must hold identically. Multiplying through by the common term $d_j^b$ and using the symmetry of the adjacency matrix yield

$$\sum_{i=1}^{N}\left(\frac{W_{ij}}{d_j^a}-\frac{W_{ij}}{d_i^a}\right)=\sum_{i=1}^{N}\frac{W_{ij}(d_i^a-d_j^a)}{d_j^a d_i^a}=0. \tag{22}$$

As in the previous case, the robustness condition (C3) implies that

$$d_i^a-d_j^a=0, \tag{23}$$

for all pairs $(i,j)$ and we conclude that mass conservation and robustness imply that $a=0$. Taken together conditions (C1), (C2) and (C3) imply that $a=b=0$ must follow. □

## Graph Laplacian correction for varying volumes

If the ROIs (nodes) have different volumes $\boldsymbol{\nu}=(\nu_1,\ldots,\nu_n)$, then the condition for the conservation of mass (C1) has to be modified. Indeed the total mass is now $P=\boldsymbol{\nu}\cdot\mathbf{p}$. Enforcing $\dot{P}=0$ in (8), implies

$$\boldsymbol{\nu}\cdot\mathcal{L}=\mathbf{0}. \tag{24}$$

This condition can easily be achieved by choosing

$$\mathcal{L}=\rho\mathbf{L}_V=\rho\nu\mathbf{V}^{-1}\mathbf{L}, \tag{25}$$

where $\mathbf{V} = \mathrm{diag}(\boldsymbol{\nu})$ and $\nu$ is a characteristic volume (average volume for instance). We note that this modified graph Laplacian is not symmetric, but that Fick's condition is still satisfied ($\mathbf{L}_V \cdot \mathbf{1} = \mathbf{0}$) since diffusion takes place when a concentration gradient is established, independently of the ROIs' volume. We also note that the multiplication of the standard Laplacian on the left by a diagonal matrix has been used to define regions of vulnerability [6, 7], hence assuming that diffusion takes place differently in different ROIs. Mathematically, it is the same operation but its interpretation in term of volumes or vulnerability is different and corresponds to different modeling choices (i.e. if we insist on mass conservation then mass should be conserved and interpreting $\mathbf{V}^{-1}$ as vulnerability precludes mass conservation).

## References


[1] J. Weickenmeier, E. Kuhl, and A. Goriely, "Multiphysics of prionlike diseases: Progression and atrophy," *Physical review letters*, vol. 121, no. 15, p. 158101, 2018.

[2] J. Weickenmeier, M. Jucker, A. Goriely, and E. Kuhl, "A physics-based model explains the prion-like features of neurodegeneration in alzheimer's disease, parkinson's disease, and amyotrophic lateral sclerosis," *Journal of the Mechanics and Physics of Solids*, vol. 124, pp. 264–281, 2019.

[3] S. Fornari, A. Schäfer, A. Goriely, and E. Kuhl, "Prion-like spreading of alzheimer's disease within the brain's connectome," *Interface R. Society*, 2019.

[4] A. Schäfer, E. Mormino, and E. Kuhl, "Network Diffusion Modeling Explains Longitudinal Tau PET Data," *Front. Neurosci.*, vol. 14, p. 1370, 2020.

[5] A. Goriely, E. Kuhl, and C. Bick, "Neuronal oscillations on evolving networks: dynamics, damage, degradation, decline, dementia, and death," *Physical review letters*, vol. 125, no. 12, p. 128102, 2020.

[6] M. X. Henderson, E. J. Cornblath, A. Darwich, B. Zhang, H. Brown, R. J. Gathagan, R. M. Sandler, D. S. Bassett, J. Q. Trojanowski, and V. M. Lee, "Spread of $\alpha$-synuclein pathology through the brain connectome is modulated by selective vulnerability and predicted by network analysis," *Nature neuroscience*, vol. 22, no. 8, p. 1248, 2019.

[7] M. X. Henderson, S. Sedor, I. McGeary, E. J. Cornblath, C. Peng, D. M. Riddle, H. L. Li, B. Zhang, H. J. Brown, M. F. Olufemi *et al.*, "Glucocerebrosidase activity modulates neuronal susceptibility to pathological $\alpha$-synuclein insult," *Neuron*, 2019.

[8] E. Toro and A. Hidalgo, "ADER finite volume schemes for nonlinear reaction–diffusion equations," *Appl. Numer. Math.*, vol. 59, no. 1, pp. 73–100, 2009.

[9] R. Hosek and J. Volek, "Discrete advection–diffusion equations on graphs: Maximum principle and finite volumes," *Appl. Math. Comput.*, vol. 361, no. 15, pp. 630–644, 2019.

[10] F. S., S. A., G. A., and E. Kuhl, "Spatially-extended nucleation-aggregation-fragmentation models for the dynamics of prion-like neurodegenerative protein-spreading in the brain and its connectome," *J. Theor. Biol.*, 2019.

[11] T. Thompson, P. Chaggar, E. Kuhl, and A. Goriely, "Protein-protein interactions in neurodegenerative diseases: a conspiracy theory," *bioRxiv*, 2020.

[12] A. Raj, A. Kuceyeski, and M. Weiner, "A network diffusion model of disease progression in dementia," *Neuron*, vol. 73, no. 6, pp. 1204–1215, 2012.

[13] F. Abdelnour, H. Voss, and A. Raj, "Network diffusion accurately models the relationship between structural and functional brain connectivity networks," *Neuroimage*, vol. 90, pp. 335–347, 2014.

[14] A. Raj, E. LoCastro, M. Weiner *et al.*, "Network diffusion model of progression predicts longitudinal patterns of atrophy and metabolism in alzheimer's disease," *Cell reports*, vol. 10, no. 3, pp. 359–369, 2015.

[15] S. Pandya, C. Mezias, and A. Raj, "Predictive model of spread of progressive supranuclear palsy using directional network diffusion," *Frontiers in neurology*, vol. 8, p. 692, 2017.

[16] S. Pandya, Y. Zeighami, B. Freeze, M. Dadar, D. Collins, and A. Raj, "Predictive model of spread of Parkinson's pathology using network diffusion," *NeuroImage*, vol. 192, pp. 178–194, 2019.
