# Supplementary material for: Braiding Braak and Braak: Staging patterns and model selection in network neurodegeneration
Source: Netw Neurosci. 2021 Nov 30;5(4):929–56. doi: 10.1162/netn_a_00208 (PMC8746141; doi:10.1162/netn_a_00208)
Supplement: Supplementary file 2 [file netn-05-929-s002.pdf]

Supplementary Information S2
Additional staging results

Additional results, deterministic streamlined connectome staging

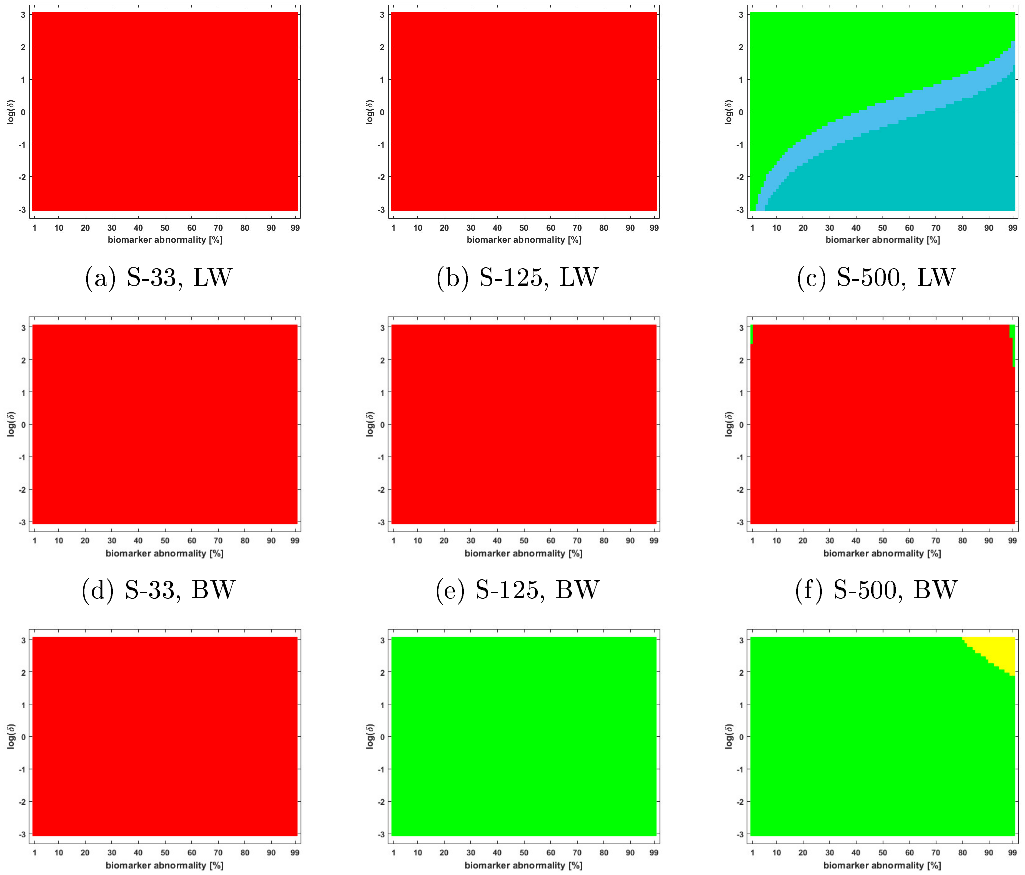

(a) S-33, LW (b) S-125, LW (c) S-500, LW

(d) S-33, BW (e) S-125, BW (f) S-500, BW

(g) S-33, DW (h) S-125, DW (i) S-500, DW

Figure 1: Braid surfaces, observed computational τP NFT staging with deterministic streamlined connectomes; diffusion dominated regime ($\ln(\beta) = 2$). Length-free (top), ballistic (middle) and diffusive (bottom) weighting schemes. The x-axis determines the biomarker abnormality threshold $1\% < T \leq 100\%$ and the y-axis corresponds to NFT aggregation rate (δ) with $-3 \leq \ln(\delta) \leq 3$.

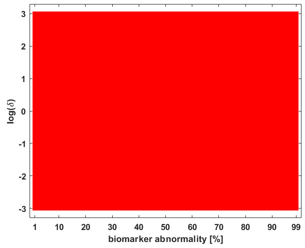

(a) S-33, LW

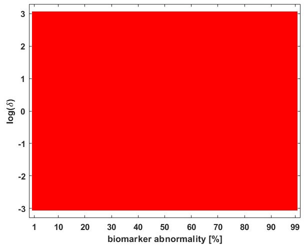

(b) S-125, LW

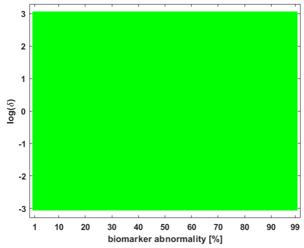

(c) S-500, LW

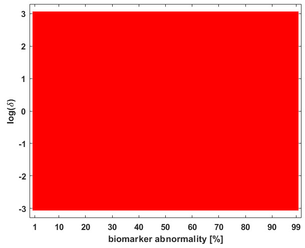

(d) S-33, BW

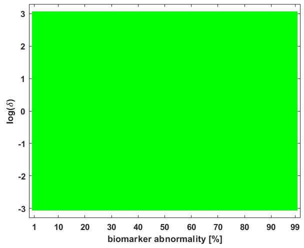

(e) S-125, BW

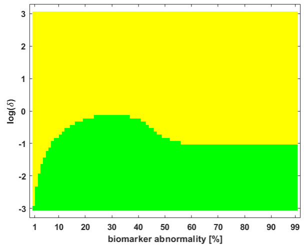

(f) S-500, BW

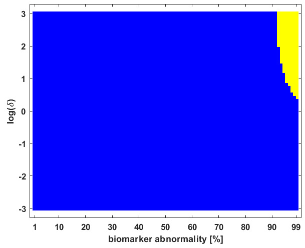

(g) S-33, DW

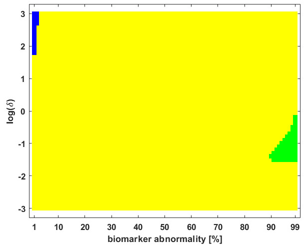

(h) S-125, DW

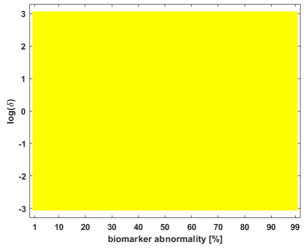

(i) S-500, DW

Figure 2: Braid surfaces, observed computational τP NFT staging with deterministic streamlined connectomes; growth dominated regime ($\ln(\beta) = -3$). Length-free (top), ballistic (middle) and diffusive (bottom) weighting schemes. The x-axis determines the biomarker abnormality threshold $1\% < T \leq 100\%$ and the y-axis corresponds to NFT aggregation rate (δ) with $-3 \leq \ln(\delta) \leq 3$.

Additional results, probabilistic streamlined connectome staging

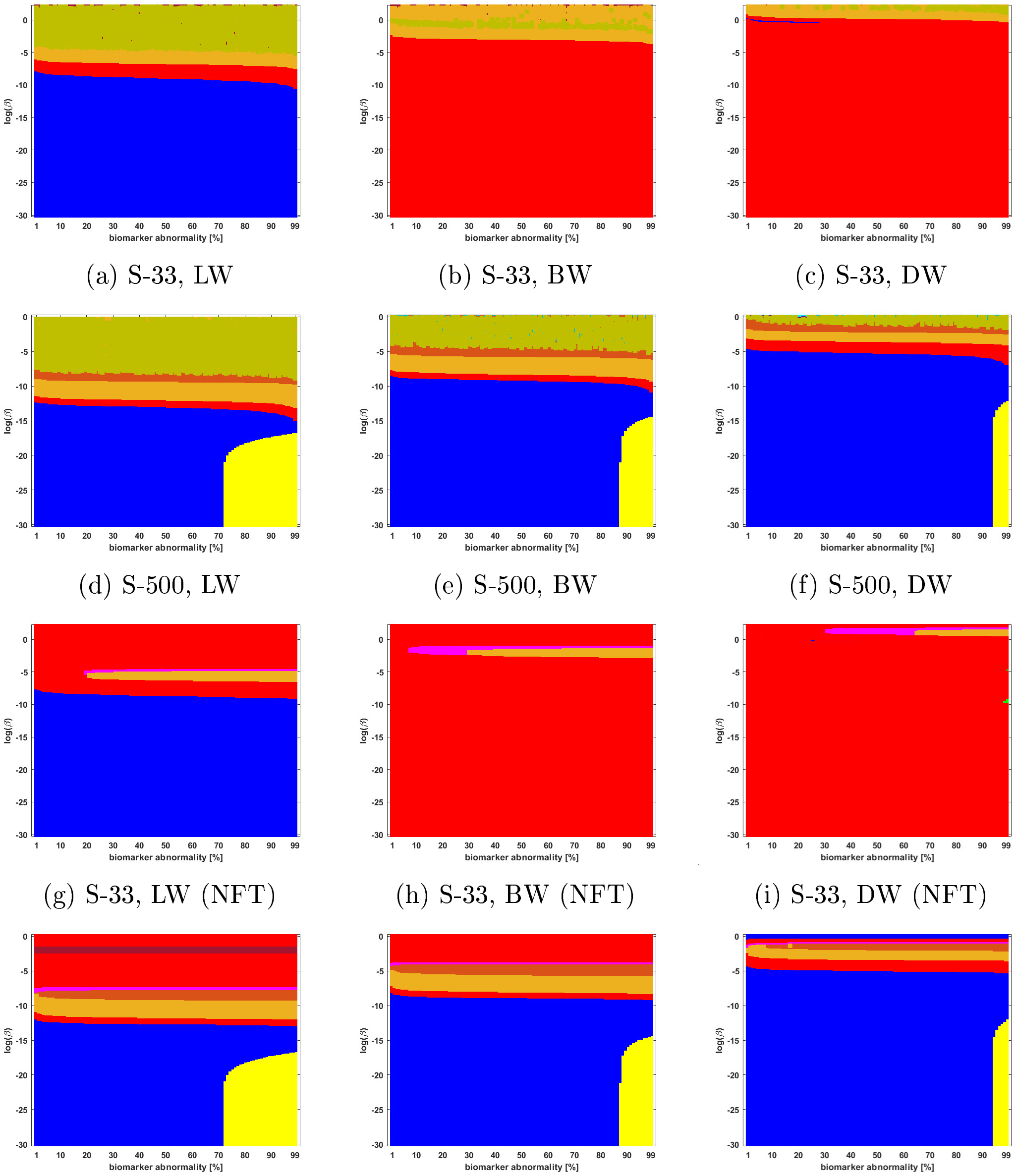

(a) S-33, LW (b) S-33, BW (c) S-33, DW

(d) S-500, LW (e) S-500, BW (f) S-500, DW

(g) S-33, LW (NFT) (h) S-33, BW (NFT) (i) S-33, DW (NFT)

(j) S-500, LW (NFT) (k) S-500, BW (NFT) (l) S-500, DW (NFT)

Figure 3: Observed computational (probabilistic) connectome τP seed staging (top two rows) and τP NFT staging (bottom two rows). Density filter thresholding at a threshold of 8×10^{-1} with biomarker abnormality $1\% \leq T \leq 100\%$ (x-axis) and $-30 \leq \ln(\beta) \leq 0$ (y-axis)

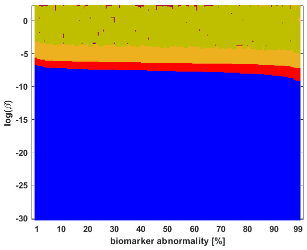

(a) S-33, LW

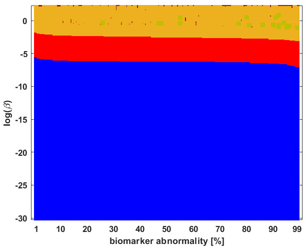

(b) S-33, BW

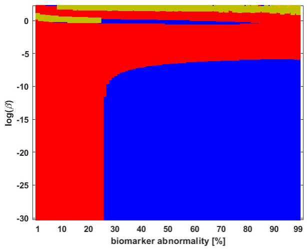

(c) S-33, DW

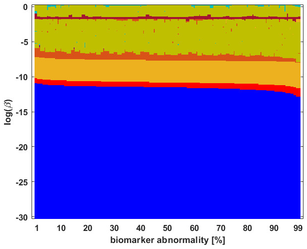

(d) S-500, LW

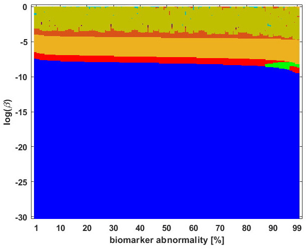

(e) S-500, BW

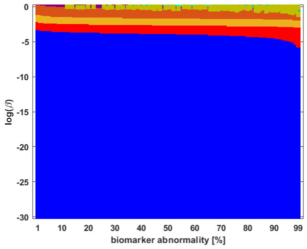

(f) S-500, DW

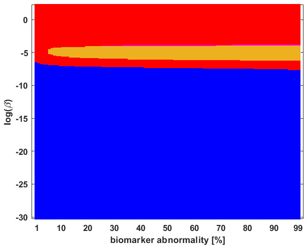

(g) S-33, LW (NFT)

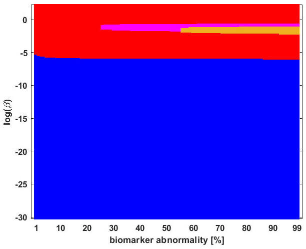

(h) S-33, BW (NFT)

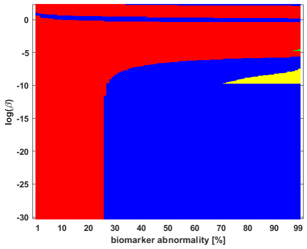

(i) S-33, DW (NFT)

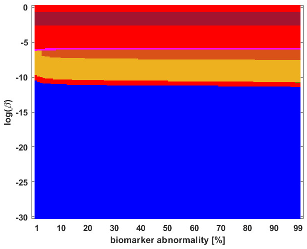

(j) S-500, LW (NFT)

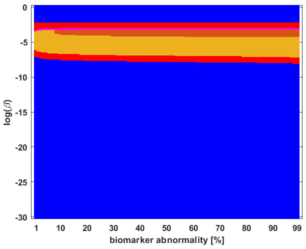

(k) S-500, BW (NFT)

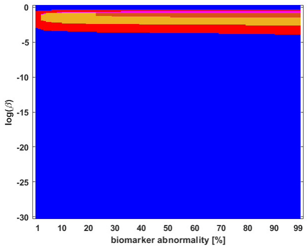

(l) S-500, DW (NFT)

Figure 4: Observed computational (probabilistic) connectome τP seed staging (top two rows) and τP NFT staging (bottom two rows). High salience skeleton at a threshold of 5×10^{-4} with biomarker abnormality $1\% \leq T \leq 100\%$ (x-axis) and $-30 \leq \ln(\beta) \leq 0$ (y-axis)

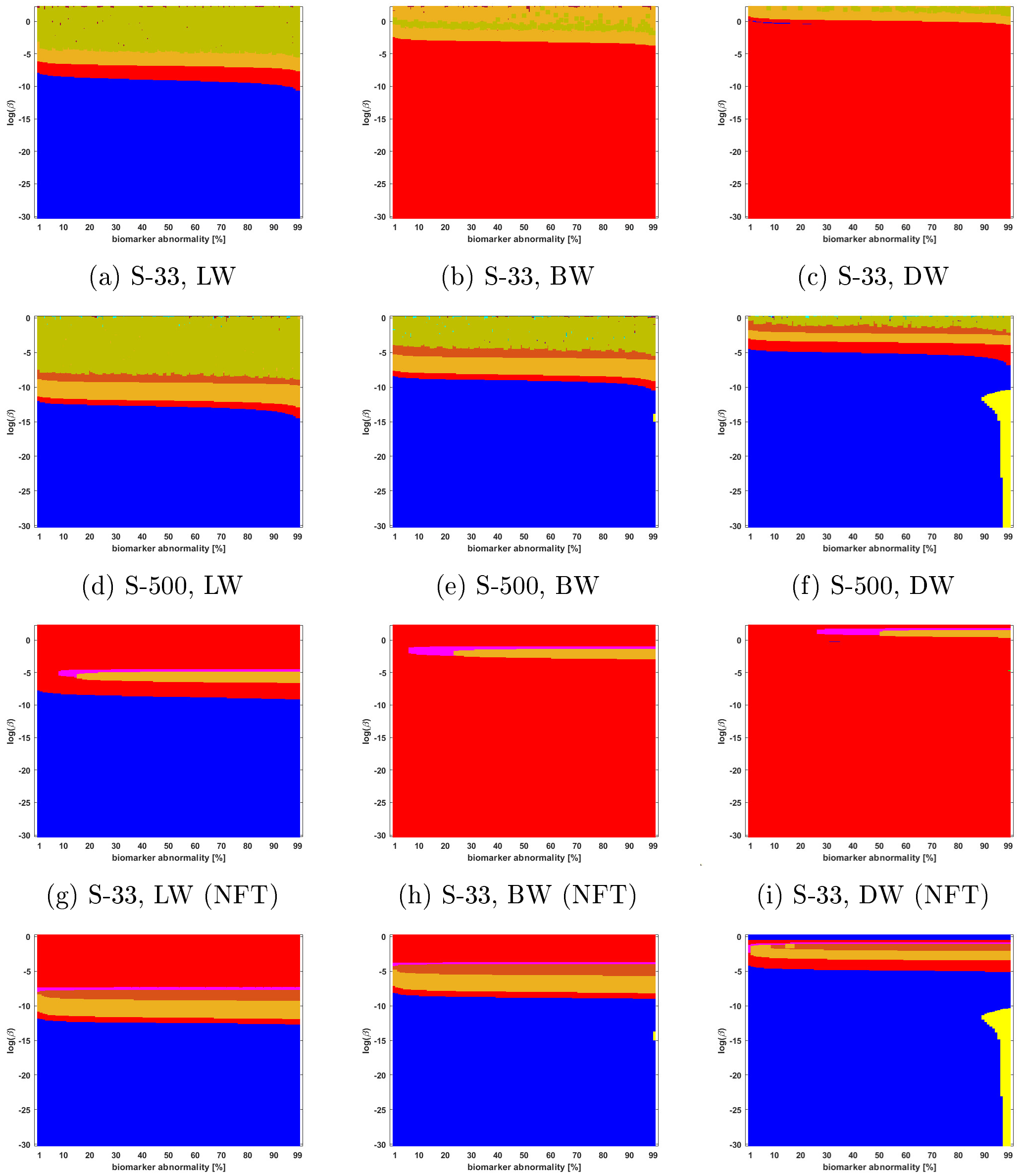

(a) S-33, LW (b) S-33, BW (c) S-33, DW

(d) S-500, LW (e) S-500, BW (f) S-500, DW

(g) S-33, LW (NFT) (h) S-33, BW (NFT) (i) S-33, DW (NFT)

(j) S-500, LW (NFT) (k) S-500, BW (NFT) (l) S-500, DW (NFT)

Figure 5: Observed computational (probabilistic) connectome τP seed staging (top two rows) and τP NFT staging (bottom two rows). Noise corrected backbone at a threshold of 1.28 with biomarker abnormality $1\% \leq T \leq 100\%$ (x-axis) and $-30 \leq \ln(\beta) \leq 0$ (y-axis)

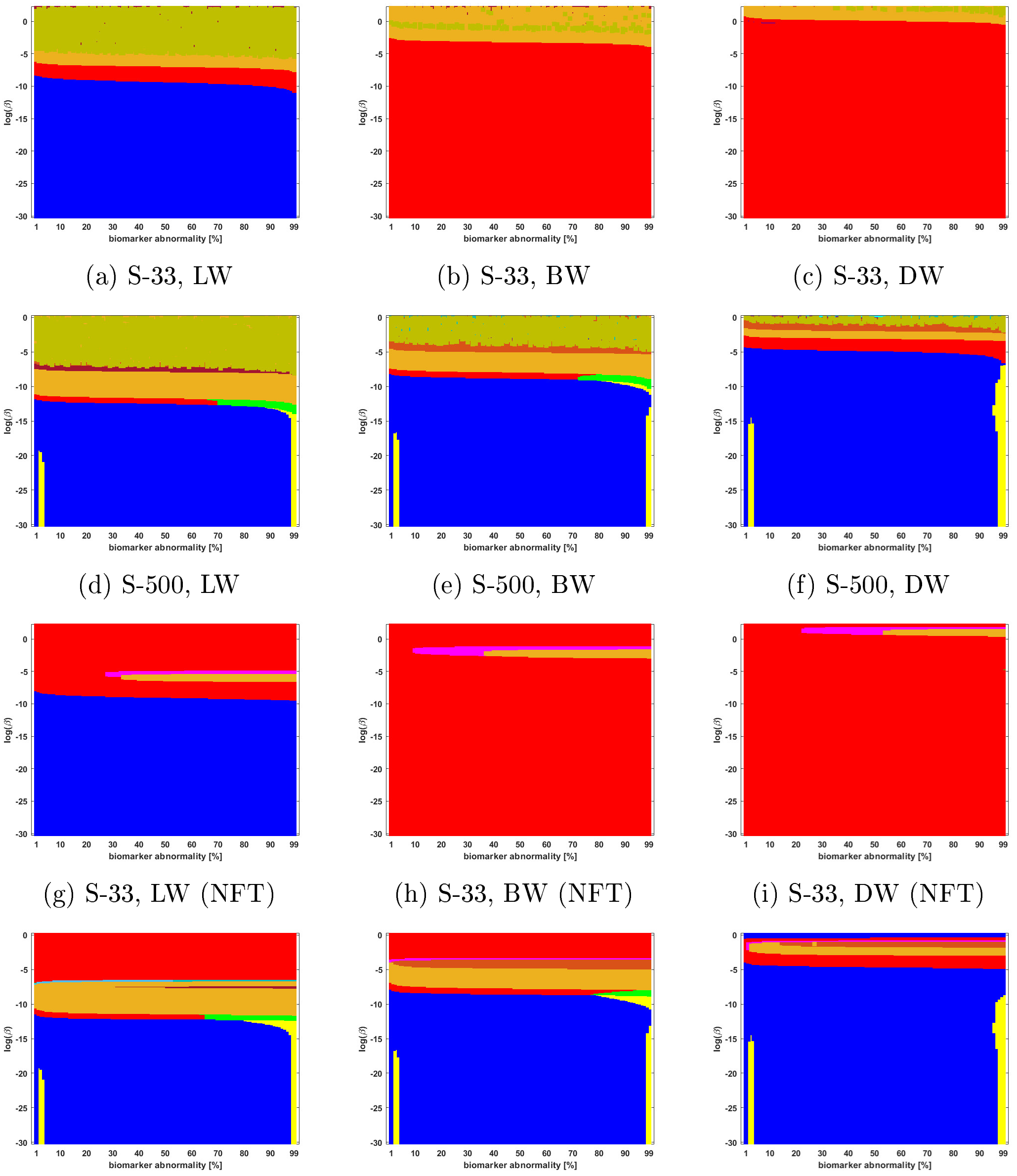

(a) S-33, LW (b) S-33, BW (c) S-33, DW

(d) S-500, LW (e) S-500, BW (f) S-500, DW

(g) S-33, LW (NFT) (h) S-33, BW (NFT) (i) S-33, DW (NFT)

(j) S-500, LW (NFT) (k) S-500, BW (NFT) (l) S-500, DW (NFT)

Figure 6: Observed computational (probabilistic) connectome τP seed staging (top two rows) and τP NFT staging (bottom two rows). Naive thresholding at a threshold of 5×10^{-3} with biomarker abnormality $1\% \leq T \leq 100\%$ (x-axis) and $-30 \leq \ln(\beta) \leq 0$ (y-axis)

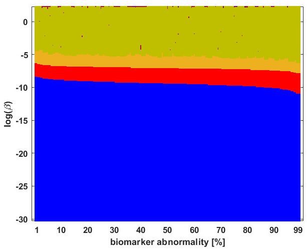

(a) S-33, LW

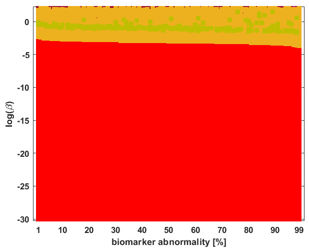

(b) S-33, BW

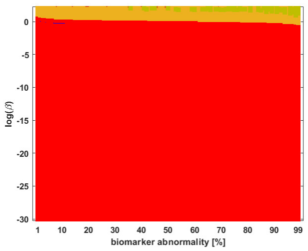

(c) S-33, DW

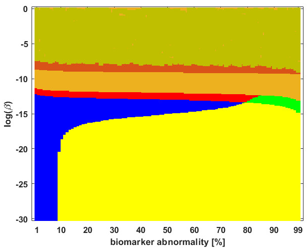

(d) S-500, LW

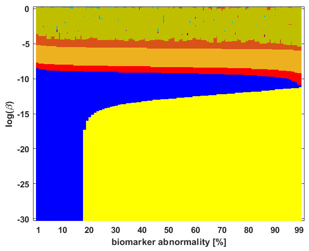

(e) S-500, BW

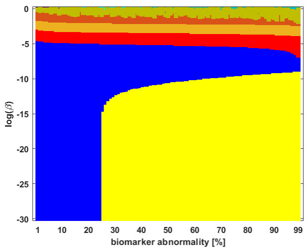

(f) S-500, DW

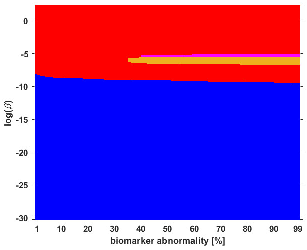

(g) S-33, LW (NFT)

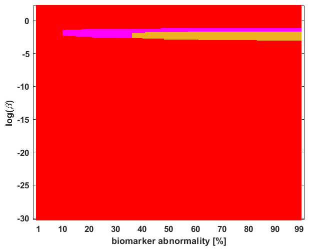

(h) S-33, BW (NFT)

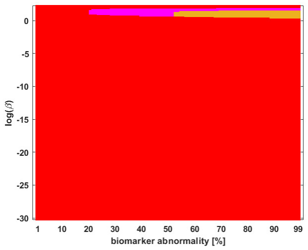

(i) S-33, DW (NFT)

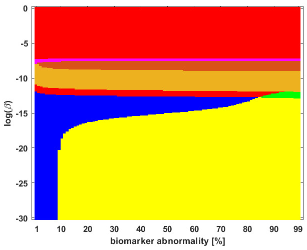

(j) S-500, LW (NFT)

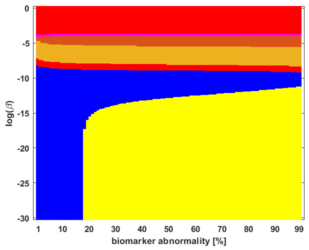

(k) S-500, BW (NFT)

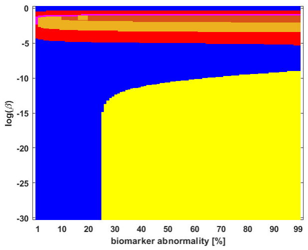

(l) S-500, DW (NFT)

Figure 7: Observed computational (probabilistic) connectome τP seed staging (top two rows) and τP NFT staging ($\delta = 1$, bottom two rows). Naive thresholding at a threshold of 1×10^{-3} with biomarker abnormality $1\% \leq T \leq 100\%$ (x-axis) and $-30 \leq \ln(\beta) \leq 0$ (y-axis)
